# Effects of Hidden Conditioning and Positive Expectations on Pain Intensity Following Spinal Manipulative Therapy: A Three‐Arm Randomized Controlled Trial

**DOI:** 10.1002/ejp.70414

**Published:** 2026-10-12

**Authors:** Helen Cristina Nogueira Carrer, Thamiris Costa de Lima, Bianca Emanuelli Saes Campanha, Steven Z. George, Felipe J. J. Reis, Deise Lilian Costa Dias, Thais Cristina Chaves

**Affiliations:** ^1^ Department of Physical Therapy Federal University of São Carlos, UFSCar São Carlos Brazil; ^2^ Postgraduate Program in Physical Therapy, Federal University of São Carlos, UFSCar São Carlos Brazil; ^3^ Departments of Orthopaedic Surgery and Population Health Science and the Duke Clinical Research Institute Duke University School of Medicine Durham North Carolina USA; ^4^ Science, Technology, and Innovation in Health, Instituto Federal de Educação Ciência e Tecnologia do Rio de Janeiro (IFRJ) Rio de Janeiro RJ Brazil; ^5^ School of Physical and Occupational Therapy, Faculty of Medicine McGill University Montreal Canada

**Keywords:** chronic pain, contextual factors, expectation, hidden conditioning, spinal manipulative therapy

## Abstract

**Background:**

Conditioning and positive expectations are potential mechanisms for pain relief. However, there is a gap in the literature on the isolated or combined impact of these factors for decreasing pain intensity.

**Methods:**

This study investigated whether a hidden conditioning procedure with a hidden lowering of the thermal noxious stimulus during a conditioning session combined with positive expectation would result in greater low back pain intensity reduction compared to positive and neutral expectations alone. All groups participating in the study received six sessions of spinal manipulative therapy (SMT). Patients (*n* = 264) with nonspecific chronic low back pain (CLBP), aged between 18 and 65 years, were randomly allocated to three groups: hidden conditioning with positive expectations (C‐PEG), positive expectations alone (PEG), or neutral expectations alone (NEUG). Participants were submitted to a conditioning test pre‐ and post‐first session of SMT, where heat pain stimuli were surreptitiously lowered only in the C‐PEG group. The primary outcomes were low back pain intensity and global perceived effect (GPE) of improvement assessed post‐first SMT session and at 6‐week (primary endpoint) and 3‐month follow‐ups. Linear mixed‐effects models were used to assess group and time effects.

**Results:**

No significant difference was found between the three groups at any of the follow‐ups for pain intensity or GPE. Positive expectations (pre‐treatment) and psychosocial variables partially mediated the relationship between baseline low back pain and disability outcomes. All groups showed a within‐group reduction in low back pain and disability at follow‐ups.

**Significance Statement:**

Conditioning and positive expectation with SMT did not improve pain intensity or GPE outcomes for participants with low back pain, when compared to positive or neutral expectations alone. These findings suggest that patients did not generalize learned associations during conditioning to low back pain intensity outcome. Regardless of treatment assignment, positive expectations and psychosocial factors played a mediating role in pain intensity, GPE, and disability outcomes.

**Trial Registration:**

ClinicalTrials.gov identifier: NCT05202704

## 1 Introduction

Substantial efforts have been directed toward identifying effective interventions for chronic low back pain (CLBP). However, both pharmacological and non‐pharmacological treatments are associated with small effects (Rizzo et al. 2025; Maher et al. 2017), which leads to continued investigation into ways to increase the effects of individual treatments. Spinal Manipulative Therapy (SMT) is a recommended intervention to manage CLBP (de Zoete et al. 2026). SMT is regarded as a passive intervention (Rhon and Deyle 2021) with small short‐term pain relief effects compared to sham treatments (de Zoete et al. 2026). The current evidence suggests that physical therapy interventions, including SMT, might have effects largely influenced by non‐specific and contextual factors (Ezzatvar et al. 2024).

The contextual effect can be conceptualized as the benefit attributable to factors related to the treatment context and is defined as a psychophysiological response arising from interactions with the therapeutic environment (Poulter et al. 2025; Cook et al. 2023; Miller and Kaptchuk 2008). Contextual effects are conceptualized as ‘placebo‐related effects’ occurring even in the absence of the inert treatment (Saueressig et al. 2024). The literature outlines various mechanisms that facilitate contextual effects impacting pain outcomes, including conditioning and expectation suggestion (Testa and Rossettini 2016; Rossettini et al. 2018). That is, pain can be modulated through learning processes, such as conditioning that establishes that associations between two events have been linked to changes in pain sensitivity (Madden et al. 2016). Furthermore, pain can be modulated by expectation suggestion, which occurs when there is an influence on the belief about a future event (Colloca and Miller 2011).

Previous mechanistic studies have examined the effects of expectancy induced by verbal suggestions related to non‐inert treatments and/or conditioning on pain intensity in patients with chronic pain (Zaworski et al. 2025, 2026; Campanha et al. 2026; Volpino et al. 2025; Malfliet et al. 2019; Kong et al. 2018; Bialosky et al. 2014). The results observed were contradictory for the effects of expectation enhancement on pain intensity following SMT. For instance, Malfliet et al. (2019) reported a significant pain reduction effect when comparing positive, neutral, and negative expectation groups, while Campanha et al. (2026) reported a significant pain decrease only when comparing both positive and neutral expectations with the negative expectation group.

To date, no study has examined a conditioning paradigm combined with induced positive expectancies related to SMT: such a design would evaluate the potential additive effects of these mechanisms on pain intensity in patients with CLBP. Therefore, this study aimed to determine whether a hidden conditioning paradigm implemented through the surreptitious reduction of a noxious stimulus following the induction of positive expectancies about SMT would produce a greater reduction in clinical low back pain intensity for those with CLBP, when compared to positive and neutral verbal suggestions alone. We hypothesized that the group receiving hidden conditioning combined with positive expectations would demonstrate a greater reduction in 6‐week clinical back pain intensity and global ratings of perceived effect. As a secondary aim, we evaluated whether expectancy and psychosocial variables mediated treatment outcomes.

## 2 Methods

### 2.1 Study Design and Ethics

This study was a randomized controlled trial with three parallel arms following the recommendations of the extension of the Consolidated Standards of Reporting Trials (CONSORT) (Juszczak et al. 2019). For all three groups, patients were submitted to four sessions of SMT in combination with assigned verbal suggestions. Group #1 was submitted to a hidden pain conditioning and positive expectation induced by verbal suggestions (C‐PEG) in which the heat noxious stimulus was surreptitiously lowered immediately after the first session of SMT. Group #2 received positive expectations induced by verbal suggestions (PEG), and group #3 received neutral expectations (NEUG) alone. There were eight stages for trial conduct: (1) Calibration to determine heat pain threshold, (2) Assessment of baseline variables and outcome measures, (3) Watching the videos: positive or neutral expectations, (4) Pre‐treatment delivery of heat noxious stimuli, (5) First session of intervention, (6) post‐treatment heat pain assessment with conditioning (C‐PEG) and without conditioning (PEG and NEUG), (7) Additional five sessions of SMT, and (8) Reassessment (6‐week and 3‐month follow‐up). Figure 1 depicts a diagram explaining the study phases.

**FIGURE 1:**
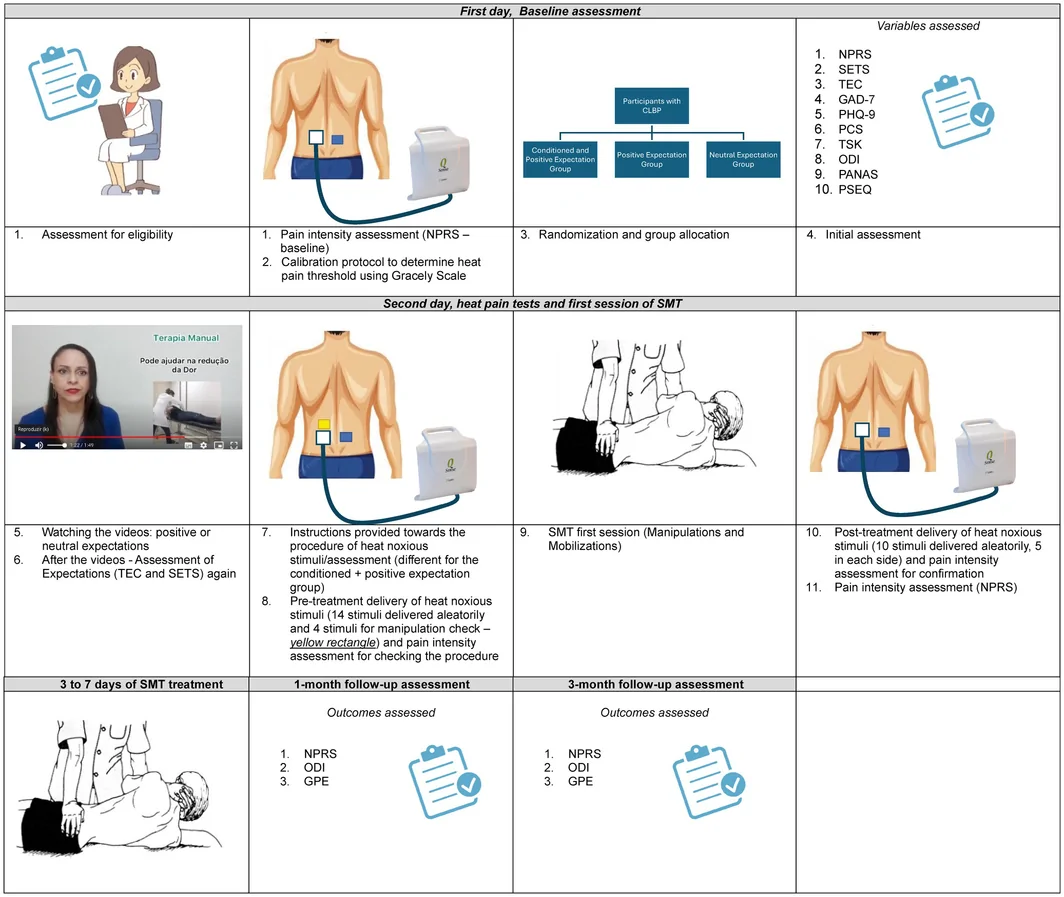
Diagram depicting the study phases. GAD‐7, General Anxiety Disorder‐7; GPE, Global Perceived Effect of Improvement; NPRS, numerical pain rating scale; ODI, Oswestry low back pain disability index; PANAS, Positive and Negative Affect Scale; PCS, pain catastrophizing scale; PHQ‐9, Patient Health Questionnaire‐9; PSEQ, pain self‐efficacy questionnaire; SETS, Standford Expectations of Treatment Scale; TEC, Treatment Expectation in Chronic pain scale; TSK, Tampa Scale for Kinesiophobia.

The mediation analysis followed the ‘A Guideline for Reporting Mediation Analyses’ (AGReMA) checklist (short form) (Lee et al. 2021). The detailed study protocol (File S1) was published (Nogueira Carrer et al. 2023) and registered prospectively on ClinicalTrials.gov (NCT05202704, https://clinicaltrials.gov/study/NCT05202704) in March 2023.

The trial was approved by the ethics committee for research involving human subjects of the Federal University of São Carlos (Process number: 52925921.5.0000.5504), and all patients provided written informed consent to participate in the study. Upon completion of the 3‐month follow‐up period, all participants who responded to the contact were informed regarding the deceptive nature of the study.

### 2.2 Participants

Participants with CLBP were recruited through local media and social networks and underwent treatment sessions and assessments at a public university–affiliated outpatient rehabilitation service in Brazil. The inclusion criteria for patients were as follows: (1) patients who reported non‐specific CLBP (in which the specific nociceptive source could not be identified, confirmed by a medical assessment) (Deyo et al. 2014) for at least 3 months duration; (2) age ranged from 18 to 60 years (Zajacova et al. 2021); (3) clinical low back pain intensity score ≥ 3 on a Numeric Pain Rating Scale (NPRS) (Chiarotto et al. 2019) asking participants regarding current pain intensity; (4) score greater than 14% on the Oswestry Disability Index (ODI) (Tavares et al. 2023), and (5) patients able to speak and understand Portuguese well to fill out the questionnaires. Patients were excluded if they met any of the following criteria: (1) Previous poor experiences with SMT through the application of a brief screening questionnaire (Colloca et al. 2008); (2) pregnancy; (3) specific LBP disorders like radiculopathy or lumbar stenosis or chronic degenerative disorders, neurological or psychiatric diseases and stroke sequelae; (4) undergoing other therapeutic interventions for chronic pain and CLBP (including surgeries) in the last three months (Clark et al. 2023); (5) presence of contraindications to SMT (e.g., fractures, severe osteoporosis, Ehlers‐Danlos syndrome) (Puentedura 2018), and (6) patients who reach the upper limit of 50°C during the calibration procedure which is the limit of safety temperature.

### 2.3 Calibration Protocol to Determine Heat Pain Threshold

The researcher in charge of the hidden pain conditioning procedure was trained for 60 h to conduct and administer the protocol. The calibration test took place in session one. Patients were submitted to a series of progressive stimuli starting at 32°C. The stimuli increased by 1°C until reaching 50°C or the participant's maximum tolerance. Each stimulus was held at each level for 5 s, including 2 s of stimulus ramp‐up and ramp‐down. The time interval between stimuli was 30 s, and subjects were invited to rate their pain intensity using the Gracely Scale (Gracely et al. 1978). This scale has 20 increasing levels of pain perception and has demonstrated good levels of reliability. Q‐sense device (Medoc) was used to assess thermal pain using a 3 cm × 3 cm thermode sensor (Egorova et al. 2015). Temperature values at which the participant reported low‐to‐moderate pain intensity (5–7 on the Gracely Scale) and severe pain intensity (14–15 on the Gracely Scale) were registered for each volunteer (Kong et al. 2018). Heat pain thresholds were obtained in two areas of the lumbar spine (blue rectangles), delimited by the same size as the thermode (Figure 1). Heat pain threshold values were set for the two areas separately. If any discrepancy in heat pain threshold between both sides was observed, the lower value was considered for the hidden pain conditioning procedure. The reference spot for delimitating the rectangles to deliver noxious stimuli was at the level of the L3 vertebra (Figure 1).

### 2.4 Randomization and Blinding

At the end of session one, after the assessment for eligibility and the calibration test to determine heat pain threshold, the participants were randomized to the study groups. After randomization, all participants completed the baseline assessment questionnaires and outcome measures. Participants were randomly assigned to three groups: (1) C‐PEG, (2) PEG, and (3) NEUG, in a 1:1:1 ratio through a computerized randomization procedure (Research Randomizer, https://www.randomizer.org/) using a block randomization model (3 blocks of 88 participants). A clinical researcher, who was not involved in the treatment or outcome assessment stages of the study, created the allocation sequence and concealed the allocation by using consecutive numbered, sealed, opaque envelopes and was in charge of the allocation assignment. The outcome assessor, patients, physiotherapist, and statistician were blinded to group allocation. Patients were not informed of the hidden conditioning with surreptitious lowering of heat pain and were not aware of the different messages (positive or neutral) delivered by the videos. The CONSORT flowchart of the study is available in Figure 2.

**FIGURE 2:**
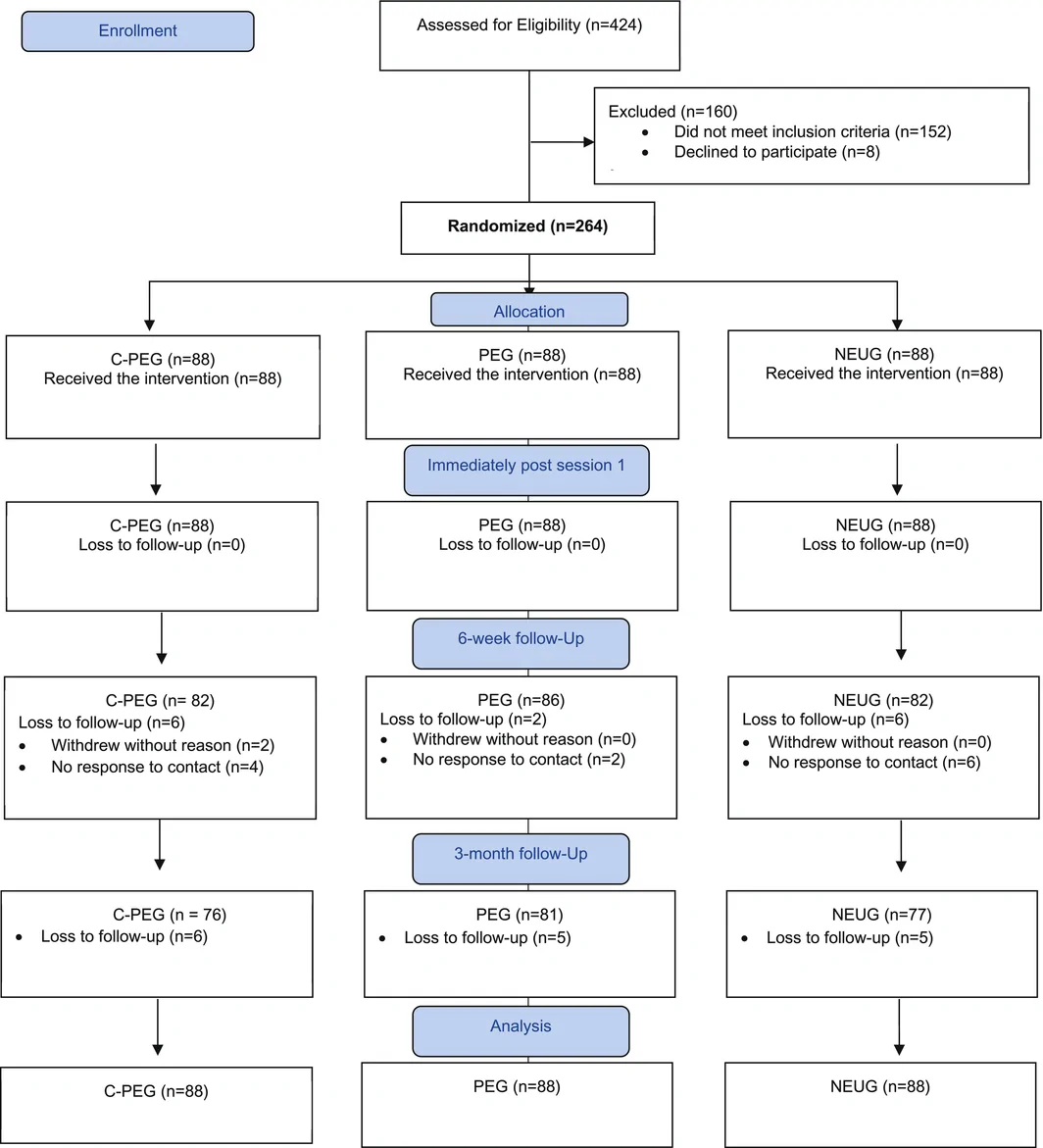
Flowchart describing study phases, participants included and loss to follow‐up. C‐PEG, conditioning + positive expectation group; NEUG, neutral expectation group; PEG, positive expectation group; SMT, Spinal Manipulative Therapy.

### 2.5 Measures

In this study, we assessed pain in three different ways: (1) Heat pain threshold for moderate/low and severe noxious stimulus (assessed with Gracely's pain rating scale) during the calibration phase of the study, (2) Pain intensity ratings (0–10) at the lower back when heat threshold was met, to confirm that participants realized the surreptitious decrease in heat noxious stimulus (see Figure 1), and (3) clinical low back pain intensity (0–10) which was assessed at the end of the first SMT session, at 6‐week (primary time point) and 3‐month follow‐ups. In this study, the reduction in pain intensity in response to a noxious heat stimulus was defined as experimental pain sensitivity. Pain intensity associated with self‐reported low back pain was defined as clinical back pain intensity.

#### 2.5.1 Primary Outcome Measures

Primary outcomes were obtained post‐first SMT session, following the 6‐week intervention (primary time point), and at the 3‐month follow‐up. This randomized trial included co‐primary outcome measures to capture different domains of improvement following SMT. Low back pain intensity was assessed by NPRS and consists of a sequence of numbers from 0 to 10, in which 0 represents ‘no pain’ and 10 represents ‘worst pain imaginable’ (Costa et al. 2008), and asks participants about current pain. The Brazilian Portuguese version showed suitable reliability (intraclass correlation coefficient [ICC] = 0.92). The minimal important change (MIC) reported for NPRS is 2 points (Salaffi et al. 2004).

Participant ratings of Global Perceived Effect (GPE) of improvement used in this trial is an 11‐point scale that ranges from −5 (‘very much worse’) through 0 (‘no change’) to +5 (‘completely recovered’), and patients were asked: ‘Compared to when this episode first started, how would you describe your low back pain these days?’. A higher score indicates a higher perception of recovery from the condition (Costa et al. 2008). The GPE Brazilian version showed acceptable reliability (ICC = 0.90) and construct validity when compared with the Roland‐Morris Questionnaire (*r* = −0.42). MIC score for GPE is 2 points (Kamper et al. 2009).

#### 2.5.2 Secondary Outcome Measure

The ODI assesses LBP‐related disability and consists of 10 items, with six response options. It ranges from 0 (absence or small amount of back pain and disability) to 5 points (extreme back pain and disability), and it has been validated in Brazilian Portuguese (Vigatto et al. 2007). The score is calculated by summing up items, with a maximum of 50 points, which can be converted to 100 points by multiplying the result by two. ODI Brazilian version showed suitable internal consistency (Cronbach's *α* = 0.87), reliability (ICC = 0.99), and acceptable construct validity when compared with the Roland Morris Questionnaire (*r* = 0.81). Ten points are the MIC value for ODI (Ostelo et al. 2008). The ODI was administered after the 6‐week intervention and at 3‐month follow‐up.

#### 2.5.3 Baseline Variables

At the baseline, additional questionnaires were administered: Patient Health Questionnaire (score range: 0–27) (PHQ‐9) (de Lima Osório et al. 2009), General Anxiety Scale (0–21) (GAD‐7) (Moreno et al. 2016), Tampa Scale for Kinesiophobia (17–68) (TSK) (Siqueira et al. 2007), Pain Catastrophizing Scale (0–52) (PCS) (Sehn et al. 2012), Positive and Negative Affect Scale (scoring: 10–50 for positive and negative affect) (PANAS) (Pires et al. 2013), and Pain Self‐Efficacy Questionnaire (0–60) (PSEQ) (Sardá et al. 2007).

Two expectation scales were administered in this study: The Standford Expectation of Treatment Scale (SETS) (Younger et al. 2012) and the Treatment Expectations in Chronic Pain Scale (TEC) (Pagé et al. 2019). The SETS has six questions with seven response options. The positive expectation domain (scoring: 3–21) must encompass the sum of items one, three, and five. The negative expectation domain score is obtained by summing up questions two, four, and six (score range: 3–21). The scale was administered pre‐treatment and soon after the hidden pain conditioning. The TEC was developed to assess ideal and predicted treatment expectations in individuals with chronic non‐cancer pain (Pagé et al. 2019). Each item is scored on a 5‐point Likert scale, from 1 for ‘strongly disagree’ to 5 for ‘strongly agree’. Total scores range from 9 to 45, with low scores indicating low expectations about treatment outcomes. Scores for ideal and predicted expectations should be calculated separately. The original study showed adequate internal consistency (Cronbach's *α* = 0.87 and 0.86 for Ideal and Predicted subscales). The authors partially confirmed the hypotheses for construct validity for the TEC scale (Pagé et al. 2019). Both scales were validated for Brazilian Portuguese and showed acceptable construct validity and reliability (ICC > 0.7) (Azevedo et al. 2026).

### 2.6 Verbal Suggestions to Induce Expectations

At the beginning of the second day of assessments, participants received specific instructions by watching videos to induce positive or neutral verbal priming. The C‐PEG and PEG received the positive suggestion instructional set, and the NEUG received the neutral instructional set. The verbal priming was administered as a video to guarantee consistency among patients. The transcription of the videos' speeches to induce positive and neutral expectations is available as File S2. The videos, with English captions, are available in full in a previous study (Campanha et al. 2026).

### 2.7 Pre‐Treatment Heat Pain Test and Assessment of Heat Pain Intensity

After watching the videos, participants were given further instructions regarding the heat pain assessment procedure. For the C‐PEG, the following instruction was provided: ‘During the test, we are going to administer heat noxious stimuli with the same intensity before and after SMT, to assess changes in your pain perception. Scientific studies showed that patients who reported a decrease in heat pain perception just after SMT are more likely to improve from low back pain after the treatment’. For the PEG and NEUG, the instruction was the following: ‘During the test, we are going to administer heat noxious stimuli with the same intensity before and after SMT to assess your heat pain intensity’.

Fourteen nociceptive stimuli of severe intensity (see Section 2.3 for details of the grade adopted to classify nociceptive stimuli) were administered at random in three locations (five stimuli for each side of the lower back) corresponding to the blue rectangles in the lower back region (Figure 1). To investigate the accuracy of the procedure and increase the unpredictability of the test, a manipulation check was adopted by administering four additional noxious thermal stimuli at random (low/moderate/severe noxious stimuli) (yellow rectangle—Figure 1). After each stimulus, the volunteers were asked to rate their perceived pain intensity using the NPRS. This protocol was based on a previous publication (Kong et al. 2018).

### 2.8 Procedures

In the first session, participants were assessed for eligibility and were submitted to the calibration test to determine the individual threshold for the heat pain assessment. In the sequence, the participants were randomized to the groups and answered the questionnaires of the study at baseline: pain intensity (NPRS), Treatment Expectations (SETS and TEC), anxiety (GAD‐7), depression (PHQ‐9), pain catastrophizing (PCS), kinesiophobia (TSK), disability (ODI), affect (PANAS) and pain self‐efficacy (PSEQ).

In the second session, participants watched videos in an attempt to induce positive or neutral expectations. Following the videos, the expectations were assessed again using SETS and TEC, and the pre‐treatment heat pain test was conducted. Immediately after the pre‐treatment heat pain assessment, participants received the first session of SMT, and the post‐treatment heat pain assessment (with hidden conditioning). Following this, clinical back pain intensity and global perceived effect (GPE) of improvement were assessed to investigate the outcome changes. The outcomes were assessed immediately after the first session (pain intensity and GPE) and at the 6‐week and 3‐month follow‐ups.

#### 2.8.1 SMT Intervention

The SMT techniques administered in the current study have been widely used in the literature (George et al. 2006; Maitland and Hengeveld 2005; Flynn et al. 2002). In this study, we consider SMT to represent any hands‐on treatment of the spine, including both manipulation and mobilization techniques applied to the spine (Rubinstein et al. 2019). Mobilizations use low‐grade velocity, small or large amplitude passive movement techniques within the patient's range of motion and control, whereas manipulation uses a high‐velocity impulse (thrust) over a short amplitude at or near the end of the passive or physiological range of motion of the spine (Rubinstein et al. 2019).

The manipulations were administered while the patient was in a supine position. The physiotherapist passively leaned over the patient to the side to be controlled, and the patient was asked to place their hands behind their head. The researcher administered a posterior and inferior push on the opposite anterior superior iliac spine (ASIS) while passively rotating the subject on the side to be manipulated. The spinal manipulations were repeated four times, with impulses oscillating between the right and left ASIS (twice on each side). In addition, mobilizations (anterior–posterior central mobilization) were applied for 1 min in each lumbar vertebra (from L5 to L1), using Grade I joint mobilizations (patients positioned in the ventral decubitus position) (Maitland and Hengeveld 2005). The same protocol was administered in all the sessions. Participants received six sessions of SMT (once per week), each one lasting 25 min. We encouraged participants to report any adverse effect (e.g., pain worsening) or need for medication during the study. The physiotherapist was blinded to group allocation. The researcher who administered the SMT is a physiotherapist with 5 years of clinical practice and certified in the manual therapy approach (280 h).

### 2.9 Post‐Treatment Heat Pain Test (Hidden Conditioning) and Assessment of Heat Pain Percetion

The patients were submitted to the first SMT session and afterward the heat pain test was performed again to conditioning participants. The hidden conditioning approach adopted in our study can be depicted as follows: in which Unconditioned Stimulus (US) is the nociceptive stimulus, Conditioned Stimulus (CS) is the SMT, and Unconditioned Response (UR) is the pain response:

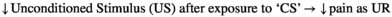




This hidden conditioning approach was based in previous studies (Watson et al. 2009; Colloca and Benedetti 2006; Klinger et al. 2007), in which the administration of the intervention (the CS) precedes the exposure to the attenuated US, and the intervention is administered once. In other words, in this paradigm, the CS (SMT in the current study) is not paired with the US to observe the conditioned response, since the CS can precede or concur with the US (Vlaeyen 2015).

In the post‐treatment heat pain assessment, a new sequence of stimuli, consisting of five low/moderate intensity stimuli (downgrading the stimuli administered in the pretreatment test to promote the hidden pain conditioning) was administered on both sides to the area marked with blue rectangles (Figure 1) for the patients in the C‐PEG. In contrast to the participants of the PEG and NEUG, stimuli with the same intensity (severe) as administered in the pre‐treatment test were delivered during the post‐treatment assessment. Each trial of noxious heat stimulation was sustained for 9 s, including 2 s of ramp‐up and ramp‐down. Immediately after each trial, the volunteers were asked to rate their perceived experimental pain sensitivity for heat noxious stimulus using NPRS for each site stimulated. The heat pain assessment tests were always carried out by the same researcher, aware of the group allocation for the C‐PEG participants (this was necessary to allow for proper administration of the hidden conditioning paradigm) but not involved in any other role in the study. The examiner was in charge of actively and surreptitiously reducing the heat pain stimuli administered to the C‐PEG group in the post‐test (after SMT), which was only possible if the examiner was aware of participants allocated to C‐PEG. Pre‐treatment and post‐treatment heat pain assessments occurred in session two, after randomization. This protocol was based on a previous publication (Kong et al. 2018).

### 2.10 Sample Size

The study was designed to identify at least a two‐point difference in clinical low back pain intensity assessed by the NPRS across groups, with an expected standard deviation of 2.5 points (Malfliet et al. 2019) when comparing C‐PEG to the other groups. For GPE, a mean difference of two points on the scale was hypothesized (Kamper et al. 2009). Sample size calculations were based on a 2‐sided *α* level of 0.05 and 80% power, yielding a required sample of 228 participants. To account for an anticipated 20% loss to follow‐up, 264 participants were enrolled (88 per group). G*Power was used to calculate sample size (G*Power v.3.0.10, University of Kiel, Germany).

### 2.11 Participant and Public Involvement in Research

We did not involve participants in the development of the study design and methods. However, we plan to involve participants to help us create leaflets to disseminate the results of the study.

### 2.12 Deviations From Protocol

There were some deviations from the initial protocol published: (1) the method to replace missing data was multiple imputation instead of last observation carried forward. Multiple imputation is preferred for missing data in randomized controlled trials (Jakobsen et al. 2017). (2) We used both RStudio and SPSS v.30 for statistical analysis. (3) In the protocol study, low back pain Improvement Expectation was included as an outcome of the study. However, we replaced the low back pain Improvement Expectation Scale with the TEC scale (Pagé et al. 2019). (4) The study protocol reported three time points: immediately after the last session, and the 6‐week and 3‐month follow‐ups. There was a mistake in the reporting of the first follow‐up, which was immediately after the first SMT session (just after the conditioning procedure). (5) In addition, in the protocol manuscript, it was reported that participants would be submitted to five sessions of SMT, but in fact, the protocol comprised six sessions of SMT.

### 2.13 Data Analysis

Analyses were performed by a statistician who received the encoded data and were based on intention‐to‐treat principles. Missing data were assumed to be missing completely at random. Little's Missing Completely at Random (MCAR) test evaluates the null hypothesis that data are missing completely at random (MCAR). The Little's MCAR test was performed using the *misty* R package (Yanagida 2023). For all the models, we observed *p* > 0.05 using the Little's MCAR test, which supported that the values in the data set were missing completely at random. Missing values in outcome variables were estimated using multiple imputation (Jakobsen et al. 2017) by chained equations after 50 replicated imputed data sets. Variables included in the multiple imputation process were: (1) a group factor, (2) a time factor, and (3) the respective outcome. Descriptive statistics were used to describe the characteristics of the participants in both treatment groups.

The primary outcomes of clinical back pain intensity and GPE, and the secondary outcome of disability were analysed using linear mixed models. Each model included participants as a random factor, and time (immediately post first SMT session, 6‐week and 3‐month follow‐ups) and treatment groups (C‐PEG, PEG and NEUG) as fixed factors, as well as the interaction between fixed factors and baseline outcome values as covariates. Repeated‐measure ANOVA was used to analyse differences in expectations between groups pre‐ and post‐videos.

The *χ* ^2^ test and Fisher's exact test were used to analyse the association between categorical variables. Fisher's exact test results were considered when the cell count showed an expected frequency less than 5 (> 20%), as Fisher's exact test has no sample size restriction. The analyses were performed using RStudio v.0.99.486 and packages ‘mice’, ‘misty’ and ‘chisq.Test’. SPSS v.22 (IBM) was used to analyse mixed models. Statistical significance was considered at a two‐sided *p* value < 0.05.

### 2.14 Mediation Analysis

Mediation analysis was conducted using structural equation modelling (SEM), which combines linear regression and factor analysis, considering both observed and unobserved variables, as well as the error associated with each variable (Mansell et al. 2016). For each single mediator model, we estimated the natural direct effect (DE), the independent variable‐mediator effect (IVME), the mediator‐outcome effect (MOE), and the indirect effect (IE) (see details in the File S3). The proportion mediated was also calculated as the fraction of total effect (TE = DE + IE) explained by the IE (IE/TE).

Pain intensity assessed at baseline was included in the models as the independent variable. As mediators, we included: positive expectation (before and after videos), negative expectation (before and after videos), and psychosocial variables (kinesiophobia, pain catastrophizing, anxiety, depression, and pain self‐efficacy). The outcomes or dependent variables considered in the study were: clinical back pain intensity just after the first SMT session and clinical back pain intensity/GPE of improvement/disability at 6‐week and 3‐month follow‐ups. The assumptions for the mediation analysis were based on previous studies (Alalawi 2026; Levenig et al. 2024; Probst et al. 2016).

For each mediation analysis, a different directed acyclic graph (DAG) was constructed (see details in the File S3). The following confounders were included in the analysis: age, pain medication, sex, and previous experience receiving SMT. All the analyses were performed using SPSS and the AMOS package. Sequential mediation analysis was conducted using bootstrapping techniques (2000 samples) and the maximum likelihood estimation method to assess indirect effects because of the non‐normal distribution of the data. The bias‐corrected bootstrap procedure to obtain confidence intervals was considered.

## 3 Results

### 3.1 Flow of Participants Through the Study

Of the 424 patients who were seeking care for CLBP, 272 were screened and eligible to receive a full clinical examination. A total of 264 participants underwent randomization (Figure 2). The first participant was enrolled in the trial in February 2022, and the last participant was enrolled in December 2023. Altogether, 100% of the participants in all three groups completed the assessments immediately post‐first SMT session. At the 6‐week follow‐up, 93% (*n* = 82) of participants in the C‐PEG, 97% (*n* = 86) in the PEG, and 93% in the NEUG completed the assessments. At the 3‐month follow‐up, 86% (*n* = 76) in the C‐PEG, 92% (*n* = 81) in the PEG, and 87.5% (*n* = 77) in the NEUG completed the assessments (Figure 2). Overall, we had a follow‐up loss of 14 participants (5%) at the 6‐week, and 16 participants at the 3‐month follow‐up (6%) (Figure 2). In the three groups, the mean frequency of sessions completed was: C‐PEG = 5.75, PEG = 5.81, NEUG = 5.53. The characteristics of the participants at baseline are presented in Table 1.

**TABLE 1:** Baseline characteristics of the study population (mean or percentages and standard deviations) obtained at baseline.

Variables	Total sample (*n* = 264)	C‐PEG (*n* = 88)	PEG (*n* = 88)	NEUG (*n* = 88)	
Age (years)	46.35 (34.90)	51.64 (58.85)	43.06 (9.10)	44.39 (9.70)	
Sex (F)	54% (*n* = 143)	52% (*n* = 46)	48% (43)	61% (*n* = 54)	*χ* ^2^(2, *n* = 264) = 0.81, *p* = 0.66
Weight (kg)	80.88 (16.97)	79.76 (16.16)	82.47 (18.19)	80.40 (16.57)	
Height (m)	168.35 (17.66)	168.71 (20.55)	168.45 (20.29)	167.89 (10.31)	
BMI (kg/m^2^)	46.51 (9.24)	46.74 (9.03)	48.28 (9.38)	44.54 (9.05)	
Education					
Incomplete elementary school	5% (*n* = 13)	4.5% (*n* = 4)	2% (*n* = 2)	8% (*n* = 7)	*χ* ^2^(2, *n* = 264) = 1.44, *p* = 0.48
Complete elementary school and high school	14% (*n* = 38)	11% (*n* = 10)	10% (*n* = 9)	10% (*n* = 9)	*χ* ^2^(2, *n* = 264) = 03, *p* = 0.98
Incomplete high school and college	47% (*n* = 125)	43% (*n* = 38)	50% (*n* = 44)	48% (*n* = 43)	*χ* ^2^(2, *n* = 264) = 0.25, *p* = 0.88
Complete college degree	35% (*n* = 98)	41% (*n* = 36)	37.5% (*n* = 33)	33% (*n* = 29)	*χ* ^2^(2, *n* = 264) = 0.37, *p* = 0.82
Received manual therapy treatment previously	24% (*n* = 64)	21.5% (*n* = 19)	31% (*n* = 21)	27% (*n* = 24)	*χ* ^2^(2, *n* = 264) = 0.29, *p* = 0.86
Previous positive experience with manual therapy and/or I am not afraid of the treatment	75% (*n* = 199)	77% (*n* = 68)	76% (*n* = 67)	73% (*n* = 64)	*χ* ^2^(2, *n* = 264) = 0.66, *p* = 0.96
No previous experience or does not have an opinion	25% (*n* = 65)	23% (*n* = 20)	31% (*n* = 21)	27% (*n* = 24)	*χ* ^2^(2, *n* = 264) = 0.19, *p* = 0.9
Analgesic comsuption	26% (70)	21.5% (*n* = 19)	28% (*n* = 25)	29.5% (*n* = 26)	*χ* ^2^(2, *n* = 264) = 0.64, *p* = 0.72
Pain Intensity (NPRS, 0–10)	5.56 (2.20)	5.45 (1.91)	5.35 (2.35)	5.89 (2.30)	
Low back pain‐related Disability (ODI) (0–100)	42.40 (9.12)	42.77 (9.12)	42.40 (7.87)	42.40 (10.08)	
Anxiety (GAD‐7, 0–21)	10.49 (6.00)	10.48 (5.49)	10.57 (6.77)	10.43 (5.71)	
Depression (PHQ‐9, 0–27)	8.96 (6.63)	8.57 (5.81)	9.09 (7.45)	9.22 (6.58)	
Catastrophizing (PCS, 0–52)	23.20 (11.81)	22.61 (12.65)	23.52 (11.15)	23.48 (11.68)	
Fear of Movement (TSK, 0–68)	41.28 (7.64)	40.85 (7.28)	41.59 (7.31)	41.41 (8.35)	
Positive Affect (PANAS, 10–50)	31.05 (8.00)	31.13 (7.79)	31.08 (8.08)	30.95 (8.20)	
Negative Affect (PANAS, 10–50)	21.76 (7.76)	21.35 (6.78)	21.51 (8.22)	22.42 (8.22)	
Pain Self‐Efficacy (PSEQ10, 0–60)	42.91 (11.55)	44.35 (11.43)	43.64 (11.98)	40.74 (11.03)	

Abbreviations: BMI, body mass index; C‐PEG, Hidden conditioning along with positive expectations group; F, female; GAD‐7, General Anxiety Disorder‐7; M, male; NEUG, neutral expectations group; NPRS, numerical pain rating scale; ODI, Oswestry low back pain disability index; PANAS, Positive and negative affect Scales; PCS, pain catastrophizing scale; PEG, positive expectations group; PHQ‐9, Patient Health Questionnaire‐9; PSEQ, Pain self‐efficacy questionnaire; TSK, Tampa Scale for Kinesiophobia.

### 3.2 Heat Pain Assessment and Pain Intensity Pre‐ and Post‐SMT

The average severe heat stimulus temperature was 41°C, while the low/moderate‐intensity heat stimulus was around 37°C for all three groups. There were significant interactions between groups and time points, considering pre‐ and post‐SMT heat pain intensity. It was found that an experimental pain decrease (estimated mean difference [MD] = −2.81; 95% CI = −3.42, −2.21) was found for C‐PEG compared to PEG, −3.51 (95% CI = −4.11, −2.9) for C‐PEG compared to NEUG, and −0.68 (95% CI = −1.29, 0.08) for PEG compared to NEUG (Table 2). The main effect of time was observed in all three groups, with a mean experimental pain decrease after SMT of −3.94 for C‐PEG, −0.95 for PEG, and −0.6 for NEUG (Table 2).

**TABLE 2:** Mean and standard deviation (SD) of the heat temperature (obtained in the calibration stage of the study) and heat pain intensity (Numerical Pain Scale, 0–10) obtained before and after the administration of Manual Therapy in the study groups, as well as change from baseline to follow‐ups (change) and estimated mean difference in the study groups in pain intensity.

Time points	C‐PEG (*n* = 88)	PEG (*n* = 88)	NEUG (*n* = 88)	Estimated mean difference C‐PEG vs. PEG (95% CI)	Estimated mean difference C‐PEG vs. NEUG (95% CI)	Estimated mean difference PEG vs. NEUG (95% CI)
**Heat temperature obtained during calibration**						
Severe temperature (°C)	41.90 (1.73)	41.88 (1.93)	41.93 (1.97)	0.02 (−1.18, 1.23)	−0.03 (−1.24, 1.17)	−0.06 (−1.26, 1.15)
Low‐Moderate temperature^a^ (°C)	37.20 (3.17)	37.43 (3.47)	37.68 (3.33)	−0.23 (−1.3, 0.85)	−0.47 (−1.55, 0.60)	−0.25 (−1.33, 0.83)
**Heat pain intensity—experimental pain (0–10)**						
Pre‐test (before manual therapy—Session 1)	7.28 (1.41)	7.11 (1.53)	7.44 (1.46)			
Post‐test (after manual therapy—Session 1)	3.33 (1.73)	6.15 (1.93)	6.84 (1.97)	−2.81* (−3.42, −2.21)	−3.51* (−4.11, −2.9)	−0.68* (−1.29, 0.08)
Change post—pre manual therapy	−3.94* (−4.27, −3.62)	−0.95* (−1.28, −0.64)	−0.60* (−0.92, −0.27)			

Abbreviations: CI, confidence interval; C‐PEG, hidden conditioning along with positive expectations group; NEUG, neutral expectations group; PEG, positive expectations group.

^a^ Used in the post‐test only for the C&PEG.

* Withingroup comparisons (*p* < 0.05), Bonferroni post hoc.

### 3.3 Treatment Expectations Pre‐ and Post‐Videos

All groups showed a mean decrease in positive expectation score on the SETS after watching the videos, with no significant difference between them (Table 3). The negative expectation score (negative SETS) also showed a significant reduction only for the C‐PEG (MD = −2.51, 95% CI: −3.47, −1.53) and NEUG (MD = −1.74, 95% CI: −2.71, −0.76) (Table 3). Between‐group comparisons revealed a significant mean difference between C‐PEG vs. PEG (MD = −0.98*, 95% CI: −1.84, −0.13), with C‐PEG showing the larger decrease in negative expectations (Table 3). Additionally, for the Q1 of the SETS which asked participants how much they agree with the following statement ‘This treatment will be completely effective’, the findings showed a significant mean difference between both C‐PEG and PEG compared to NEUG (MD = 0.29, 95% CI: 0.006, 0.58 and MD: 0.34, 95% CI: 0.05, 0.63, respectively). It was not observed changes in TEC scores within and between groups (Table 3).

**TABLE 3:** Expectations scores (mean and standard deviation and mean difference) assessed using the Standford Expectations of Treatment Scale (SETS) and Treatment Expectation in Chronic pain scale (TEC) obtained in the different stages of the study.

Phases of the study	C‐PEG (*n* = 88)	PEG (*n* = 88)	NEUG (*n* = 88)	Estimated mean difference C‐PEG vs. PEG (95% CI)	Estimated mean difference C‐PEG vs. NEUG (95% CI)	Estimated mean difference PEG vs. NEUG (95% CI)
**SETS (positive expectation, 0–21)**						
Baseline	17.72 (3.06)	18.69 (2.84)	18.15 (2.78)			
Post‐video Session 1	14.43 (5.15)	15.54 (5.13)	14.64 (4.83)	−1.10 (−2.59, 0.37)	−0.21 (−1.68, 1.25)	0.89 (−0.59, 2.37)
Change post‐video to baseline	−3.29* (−4.40, −2.17)	−3.15* (−4.28, −2.01)	−3.50* (−4.62, −2.38)			
**Q1—This treatment will be completely effective (0–7)**						
Baseline	5.89 (1.30)	6.17 (1.17)	5.97 (1.33)			
Post‐video	6.22^a^ (0.99)	6.34^a^ (0.95)	5.93 (1.16)	−0.04 (−0.33, 0.25)	0.29 (0.006, 0.58)	0.34 (0.05, 0.63)
**SETS (negative expectation, 0–21)**						
Baseline	5.30 (3.65)	4.74 (3.11)	4.96 (4.97)			
Post‐video Session 1	2.79 (3.77)	3.78 (3.59)	3.22 (3.87)	−0.98* (−1.84, −0.13)	−0.43 (−1.28, −0.42)	0.55 (−0.30, −1.41)
Change post‐video to baseline	−2.51* (−3.47, −1.53)	−0.96 (−1.94, 0.01)	−1.74* (−2.71, −0.76)			
**TEC predicted (0–45)**						
Baseline	40.87 (4.21)	41.64 (3.64)	40.86 (4.06)			
Post‐video Session 1	41.51 (3.27)	41.65 (3.67)	41.03 (5.15)	−0.14 (−1.51, 1.22)	0.47 (−0.88, 1.83)	0.61 (−0.75, 1.90)
Change post‐video to baseline	0.63 (−0.24, 1.50)	0.01 (−0.87, 0.89)	−0.17 (−1.03, 0.70)			
**TEC ideal (0–45)**						
Baseline	44.40 (1.44)	44.39 (1.79)	43.97 (2.08)			
Post‐vídeo Session 1	44.08 (2.25)	43.87 (3.67)	43.75 (3.73)	0.20 (−0.53, 0.94)	0.33 (−0.41, 1.06)	0.12 (−0.62, 0.86)
Change post‐video to baseline	−0.33 (−0.86, 0.21)	−0.52 (−1.05, 0.02)	−0.23 (−0.76, 0.31)			

Abbreviations: 95% CI, 95% confidence interval; NEUG, neutral expectations group; PEG, positive expectations group; Q1, Question 1 from the Standford Expectations of Treatment Scale (SETS).

^a^ Between group differences (C&PEG and PEG compared to NEUG), adjusting for baseline values, Bonferroni post hoc, C‐PEG, Hidden conditioning along with positive expectations group, PEG and NEUG.

* Within‐group comparisons (*p* < 0.05), Bonferroni post hoc.

### 3.4 Primary Outcomes

No interaction between groups and time was observed for clinical back pain intensity, and GPE in the three follow‐ups of the study (post‐first SMT session, 6‐week and 3‐month follow‐ups). However, the main effect of time was shown across all three groups for clinical back pain intensity in all the follow‐ups (Table 4). For within‐group changes, the mean decrease exceeded the MIC for C‐PEG immediately post‐first SMT session (mean = −2.2, 95% CI: −2.94, −1.46) and at the 6‐week follow‐up (mean = −2.17, 95% CI: −2.90, −1.44). In the PEG, the decrease exceeded the MIC only post SMT session one (mean = −2.06, 95% CI: −2.94, −1.4), and for the NEUG only at the 6‐week follow‐up (mean = −2.31, 95% CI: −3.06, −1.56) (Table 4). The NEUG showed a significant decrease in GPE of improvement, but it was not clinically relevant (Table 4). All the groups showed a consistent score in GPE of improvement greater than 2 units at 6‐week and 3‐month follow‐ups (Table 4).

**TABLE 4:** Mean (standard deviation) of pain intensity, global perception of improvement (GPE of Improvement), and Disability (Oswestry Disability Index) at study time points.

Time points of the study	C‐PEG (*n* = 88)	PEG (*n* = 88)	NEUG (*n* = 88)	Estimated mean difference C‐PEG vs. PEG (95% CI)	Estimated mean difference C‐PEG vs. NEUG (95% CI)	Estimated mean difference PEG vs. NEUG (95% CI)
**Clinical low back pain intensity**						
Baseline	5.56 (1.91)	5.56 (2.35)	5.56 (2.30)			
Post calibration	5.15 (2.42)	5.08 (2.52)	5.15 (2.19)			
Post manual therapy/conditioning Session 1	3.35 (2.23)	3.63 (2.51)	3.57 (2.69)	−0.13 (−0.86, 0.59)	−0.22 (−0.95, 0.50)	0.08 (−0.64, 0.82)
Change after SMT Session 1 to baseline	−2.2* (−2.94, −1.46)	−2.06* (−2.94, −1.4)	−1.98* (−2.73, −1.23)			
6‐week follow‐up	3.38 (2.52)	3.63 (2.56)	3.24 (2.83)	−0.25 (−0.97, 0.48)	0.14 (−0.59, 0.86)	−0.38 (−0.34, 1.12)
Change 6‐week follow‐up to baseline	−2.17* (−2.90, −1.44)	−1.92* (−2.67, −1.17)	−2.31* (−3.06, −1.56)			
3‐month Follow‐up	3.70 (2.65)	3.71 (2.56)	3.83 (2.83)	−0.01 (−0.74, 0.71)	−0.12 (−0.85, 0.59)	−0.11 (−0.84, 0.61)
Change 3‐month follow‐up to baseline	−1.85* (−2.59, −1.11)	−1.84* (−2.58, −1.09)	−1.73* (−2.47, −0.98)			
**Global perceived effect of improvement**						
6‐week follow‐up	2.79 (1.72)	2.69 (1.77)	2.86 (2.15)	0.11 (−0.59, 0.81)	−0.07 (−0.77, 0.63)	−0.17 (− 0.88, 0.53)
3‐month follow‐up	2.47 (1.94)	2.43 (1.73)	2.26 (2.40)	0.04 (−0.66, −0.74)	0.21 (−0.91, 0.48)	0.17 (−0.87, 0.53)
Change 3‐month to 6‐week follow‐up	−0.32 (−0.04, 0.68)	−0.25 (−0.62, 0.11)	−0.60* (−0.96, −0.24)			
**Low back pain‐related disability**						
Baseline	42.77 (9.12)	42.40 (7.87)	42.40 (10.08)			
6‐week follow‐up	19.47 (11.26)	20.53 (11.75)	20.12 (15.38)	−1.05 (−4.74, 2.63)	−0.64 (−4.29, 3.00)	0.41 (−3.30, 4.12)
Change 6‐week follow‐up to baseline	−23.29* (−26.56–20.34)	−21.86* (−24.93, −18.84)	−22.28* (−25.26, −19.29)			
3‐month follow‐up	19.31 (11.94)	20.98 (14.49)	20.28 (14.91)	−1.67 (−5,36, 2.01)	−0.96 (−4.61, 2.67)	0.71 (−3.00, 4.42)
Change 3‐month follow‐up to baseline	−23.46* (−26.42, −20.50)	−21.41* (−24.47, −18.35)	−22.12* (−25.11, −19.13)			

Abbreviations: CI, confidence interval; C‐PEG, hidden conditioning along with positive expectations group; NEUG, neutral expectations group; PEG, positive expectations group; SMT, spinal manipulative therapy.

* Within group comparisons (*p* < 0.05), Bonferroni post hoc.

### 3.5 Secondary Outcome

No interaction between groups and time was observed for low back pain‐related disability. However, the main effect of time was shown across all three groups for low back pain‐related disability (Table 4). All groups showed a within‐group mean difference decrease larger than 20 units in the ODI score (C‐PEG, MD = −21.86, 95% CI: −24.93, −18.84, PEG, MD = −22.28, 95% CI: −25.26, −19.29, and NEUG: MD = −22.28, 95% CI: −25.26, −19.29) (Table 4).

### 3.6 Adverse Effects

Seventeen participants in the C‐PEG reported analgesic medication intake during the trial due to pain worsening. In the PEG and NEUG, 20 participants reported the use of medication. Most of the participants reported intake of nonsteroidal anti‐inflammatory drugs. Three participants in the C‐PEG reported using opioids during the study, and four participants in the PEG.

### 3.7 Mediation Analysis

We carried out the mediation analysis with the whole sample without including treatment assignment in the model (*n* = 264) because no between‐group difference was observed. The mediation analysis showed a partial mediation effect of positive expectation on the relationship between baseline clinical back pain intensity and disability (6‐week and 3‐month follow‐ups) for patients with CLBP submitted to SMT intervention (Table 5). Partial mediation occurs when there is a previous direct effect between independent and dependent variables, and the mediator explains only partially the effect of the independent variable on the dependent variable. The proportion of the TE mediated by positive expectation on disability was 10% and 12%. A full mediation effect of the positive expectation on the relationship between baseline pain and GPE of improvement was also observed (Table 5), which means an indirect effect between baseline clinical back pain intensity and GPE of improvement that was not presented as a direct relationship. The negative expectation did not show any mediation effect on the models investigated in the study (Table 5).

**TABLE 5:** Regression estimates of the mediation analysis, including *baseline* positive and negative expectations (SETS) as mediators and global perceived effect of improvement and disability outcomes as dependent variables (*n* = 264).

IVME = Independent Variable Mediator Effect (a path)	Estimate^a^	SE	*p*	95% CI	BCI *p*
MOE = Mediator‐Outcome Effect (b path)					
DE = Direct Effect (c path)					
IE = Indirect Effect (c' path)					
**Baseline positive expectation as mediator and baseline pain as independent variable**					
IVME: pain baseline → positive Expectation	0.22	0.07	0.003	0.08, 0.37	0.002
DE: pain baseline → pain immediately post‐SMT	0.53	0.05	< 0.001	0.42, 0.64	< 0.001
MOE: positive expectation → pain immediately post‐SMT	−0.02	0.05	0.62	−0.10, 0.06	0.58
IE: pain baseline → positive Expectation → pain immediately post‐SMT	−0.005	NA	NA	−0.03, 0.01	0.49
*No mediation effect (only IVME and DE)*					
DE: pain baseline → pain 6‐week FU	0.48	0.06	< 0.001	0.36, 0.60	< 0.001
MOE: positive expectation → pain 6‐week FU	−0.02	0.05	0.68	‐,11, 0.06	0.62
IE: pain baseline → positive Expectation → pain 6‐week FU	−0.004	NA	NA	−0.03, 0.01	0.55
*No mediation effect (only DE)*					
DE: pain baseline → pain 3‐month FU	0.44	0.06	< 0.001	0.31, 0.56	< 0.001
MOE: positive Expectation → pain 3‐month FU	−0.05	0.05	0.34	0.15, 0.04	0.32
IE: pain baseline → positive expectation → pain 3‐month FU	−0.01	NA	NA	−0.04, 0.008	0.23
*No mediation effect (only DE)*					
DE: pain baseline → disability 6‐week FU	1.3	0.3	< 0.001	0.63, 1.9	< 0.001
MOE: positive Expectation → disability 6‐week FU	−0.56	0.25	0.03	−1.08, −0.06	0.03
IE: pain baseline → positive expectation → disability 6‐week FU	−0.12	NA	NA	−0.32, −0.02	0.02
*Partial mediation —* Proportion mediated	10% (95% CI: 0.05, 0.15)				
DE: pain baseline → disability 3‐month FU	1.6	0.34	< 0.001	0.99, 2.3	< 0.001
MOE: positive expectation → disability 3‐month FU	−0.72	0.27	0.01	−1.31, −0.19	0.01
IE: pain baseline → positive expectation → disability 3‐month FU	−0.16	NA	NA	−0.40, −0.04	0.006
*Partial mediation —* Proportion mediated	12% (95% CI: 0.07, 0.16)				
DE: pain baseline → GPE 6‐week FU	−0.008	0.05	0.86	−0.12, 0.09	0.85
MOE: positive expectation → GPE 6‐week FU	0.09	0.04	0.01	0.008, 0.19	0.03
IE: pain baseline → positive Expectation → GPE 6‐week FU	0.02	NA	NA	0.003, 0.05	0.01
*Full mediation*					
DE: pain baseline → GPE 3‐month FU	−0.07	0.05	0.16	−0.18, 0.03	0.18
MOE: positive expectation → GPE 3‐month FU	0.09	0.04	0.04	0.009, 0.18	0.03
IE: pain baseline → positive expectation → GPE 3‐month FU	0.02	NA	NA	0.003, 0.06	0.02
*Full mediation*					
**Baseline negative expectation as mediator and baseline pain as independent variable**					
IVME: pain baseline → negative expectation	0.08	0.09	0.34	−0.09, 0.28	0.34
DE: pain baseline → pain immediately post‐SMT	0.53	0.05	< 0.001	0.42, 0.63	< 0.001
MOE: negative expectation → pain immediately post‐SMT	−0.02	0.04	0.57	−0.10, 0.05	0.56
IE: pain baseline → negative expectation → pain immediately post‐SMT	−0.002	NA	NA	−0.02, 0.004	0.35
*No mediation effect (only DE)*					
DE: pain baseline → pain 6‐week FU	0.48	0.06	< 0.001	0.35, 0.59	< 0.001
MOE: negative expectation → pain 6‐week FU	0.04	0.04	0.32	−0.05, 0.12	0.37
IE: pain baseline → negative expectation → pain 6‐week FU	0.003	NA	NA	−0.004, 0.03	0.27
*No mediation effect (only DE)*					
DE: pain baseline → pain 3‐month FU	0.43	0.06	< 0.001	0.31, 0.54	< 0.001
MOE: negative expectation → pain 3‐month FU	0.01	0.04	0.79	−0.08, 0.11	0.82
IE: pain baseline → negative expectation → pain 3‐month FU	0.001	NA	NA	−0.008, 0.02	0.57
*No mediation effect (only DE)*					
DE: pain baseline → negative expectation	0.08	0.09	0.34	−0.09, 0.27	0.36
DE: pain baseline → disability 6‐week FU	1.15	0.32	< 0.001	0.46, 1.8	0.001
MOE: negative expectation → disability 6‐week FU	0.25	0.22	0.24	−0.19, 0.69	0.26
IE: pain baseline → negative expectation → disability 6‐week FU	0.02	NA	NA	−0.02, 0.15	0.22
*No mediation (only DE)*					
DE: pain baseline → disability 3‐month follow‐up	1.48	0.34	< 0.001	0.82, 2.17	0.001
MOE: negative expectation → disability 3‐month FU	0.14	0.23	0.54	−0.31, 0.65	0.54
IE: pain baseline → negative expectation → disability 3‐month FU	0.01	NA	NA	−0.02, 0.14	0.33
*No mediation (only DE)*					
DE: pain baseline → GPE 6‐week FU	0.01	0.05	0.77	−0.09, 0.11	0.79
MOE: negative expectation → GPE 6‐week FU	−0.01	0.03	0.78	−0.07, 0.05	0.73
IE: pain baseline → negative expectation → GPE 6‐week FU	−0.001	NA	NA	−0.01, 0.004	0.44
*No mediation effect*					
DE: pain baseline → GPE 3‐month follow‐up	−0.05	0.05	0.33	−0.17, 0.06	0.36
MOE: negative expectation → GPE 3‐month follow‐up	−0.05	0.04	0.19	−0.12, 0.03	0.25
IE: pain baseline → negative expectation → GPE 3‐month FU	−0.004	NA	NA	−0.03, 0.003	0.23
*No mediation effect*					

Abbreviations: BCI, bootstrapped confidence interval; FU, follow‐up; GPE, global perceived effect; NA, not applicable; SE, standard error; SETS, Stanford Treatment Expectation Scale; SMT, spinal manipulative therapy.

^a^ Unstandardized parameter estimates.

We also investigated the mediation effect of positive and negative expectations after the videos on the relationship between baseline clinical back pain intensity and the outcomes of the study. Negative or positive expectations assessed after watching the videos did not mediate the relationship between baseline clinical back pain intensity (independent variable) and the outcomes of the study (dependent variables) after SMT intervention (Table 6).

**TABLE 6:** Regression estimates of the mediation analysis, including positive and negative Expectations (SETS) *post‐videos* as mediators and global perceived effect (GPE) of improvement, disability, and pain intensity outcomes as dependent variables (*n* = 264).

IVME = Independent Variable Mediator Effect (a path)	Estimate^a^	SE	*p*	95% CI	BCI *p*
MOE = Mediator‐Outcome Effect (b path)					
DE = Direct Effect (c path)					
IE = Indirect Effect (c' path)					
**Positive expectation *—*post‐video as mediator and baseline pain as independent variable**					
IVME: pain baseline → positive expectation post‐video	0.08	0.13	0.47	−0.15, 0.37	0.45
DE: pain baseline → pain immediately post‐SMT	0.53	0.06	< 0.001	0.42, 0.64	< 0.001
MOE: positive expectation → pain immediately post‐SMT	0.000	0.03	0.99	−0.05, 0.05	0.95
IE: pain baseline → positive expectation → pain immediately post‐SMT	−0.005	NA	NA	−0.03, −0.01	0.48
*No mediation effect (only DE)*					
DE: pain baseline → pain 6‐week FU	0.48	0.06	< 0.001	0.36, 0.6	< 0.001
MOE: positive expectation → pain 6‐week FU	0.02	0.03	0.49	−0.04, 0.07	0.51
IE: pain baseline → positive expectation → pain 6‐week FU	−0.004	NA	NA	−0.03, 0.01	0.56
*No mediation effect (only DE)*					
DE: pain baseline → pain 3‐month FU	0.44	0.06	< 0.001	0.31, 0.6	< 0.001
MOE: positive expectation → pain 3‐month FU	0.05	0.03	0.11	−0.009, 0.10	0.10
IE: pain baseline → positive expectation → pain 3‐month FU	−0.01	NA	NA	−0.04, 0.008	0.21
*No mediation effect (only DE)*					
DE: pain baseline → disability 6‐week FU	1.17	0.32	< 0.001	0.52, 1.84	0.001
MOE positive expectation → disability 6‐week FU	−0.006	0.15	0.97	−0.31, 0.30	0.98
IE: pain baseline → positive expectation → disability 6‐week FU	−0.001	NA	NA	−0.06, 0.05	0.89
*No mediation effect (only DE)*					
DE: pain baseline → disability 3‐month FU	1.5	0.34	< 0.001	0.85, 2.17	0.001
MOE: positive expectation → disability 3‐month FU	−0.06	0.16	0.72	−0.35, 0.28	0.76
IE: pain baseline → positive expectation → disability 3‐month FU	−0.004	NA	NA	−0.11, 0.03	0.50
*No mediation effect (only DE)*					
DE: pain baseline → GPE 6‐week FU	0.01	0.05	0.74	−0.08, 0.12	0.77
MOE: positive expectation → GPE 6‐week FU	−0.03	0.02	0.18	−0.07, 0.02	0.23
IE: pain baseline → positive expectation → GPE 6‐week FU	−0.003	NA	NA	−0.02, 0.004	0.31
*No mediation effect*					
DE: pain baseline → GPE 3‐month FU	−0.05	0.05	0.32	−0.17, 0.05	0.35
MOE: positive expectation → GPE 3‐month FU	−0.03	0.02	0.21	−0.08, 0.02	0.22
IE: pain baseline → positive expectation → GPE 3‐month FU	−0.003	NA	NA	−0.02, 0.004	0.30
*No mediation effect*					
**Negative expectation *—* post‐video as mediator and baseline pain as independent variable**					
IVME: pain baseline → negative expectation	0.06	0.10	0.55	−0.15, 0.26	0.58
DE: pain baseline → pain immediately post‐SMT	0.53	0.06	< 0.001	0.42, 0.63	< 0.001
MOE: negative expectation → pain immediately post‐SMT	−0.006	0.03	0.84	−0.06, 0.05	0.87
IE: pain baseline → negative expectation → pain immediately post‐SMT	−0.002	NA	NA	−0.02, 0.004	0.34
*No mediation effect (only DE)*					
DE: pain baseline → pain 6‐week FU	0.48	0.06	< 0.001	0.35, 0.58	< 0.001
MOE: negative expectation → pain 6‐week FU	0.04	0.04	0.30	−0.03, 0.11	0.27
IE: pain baseline → negative expectation → pain 6‐week FU	0.003	NA	NA	−0.003, 0.03	0.25
*No mediation effect (only DE)*					
DE: pain baseline → pain 3‐month FU	0.43	0.06	< 0.001	0.31, 0.54	< 0.001
MOE: negative expectation → pain 3‐month FU	0.06	0.04	0.09	−0.02, 0.15	0.11
IE: pain baseline → negative expectation → pain 3‐month FU	0.001	NA	NA	−0.008, 0.02	0.54
*No mediation effect (only DE)*					
DE: pain baseline → disability 6‐week FU	1.16	0.32	< 0.001	0.5, 1.83	0.001
MOE: negative expectation → disability 6‐week FU	0.18	0.19	0.35	−0.17, 0.59	0.32
IE: pain baseline → negative expectation → disability 6‐week FU	0.01	NA	NA	−0.02, 0.13	0.37
*No mediation effect (only DE)*					
DE: pain baseline → disability 3‐month FU	1.48	0.34	< 0.001	0.83, 2.17	0.001
MOE: negative expectation → disability 3‐month FU	0.22	0.20	0.27	−0.17, 0.63	0.27
IE: pain baseline → negative expectation → disability 3‐month FU	0.01	NA	NA	−0.02, 0.14	0.37
*No mediation effect (only DE)*					
DE: pain baseline → GPE 6‐week FU	0.01	0.05	0.78	−0.09, 0.12	0.82
MOE: negative expectation → GPE 6‐week FU	0.001	0.03	0.97	−0.05, 0.04	0.94
IE: pain baseline → negative expectation → GPE 6‐week FU	< 0.000	NA	NA	−0.006, 0.007	0.93
*No mediation effect*					
DE: pain baseline → GPE 3‐month follow‐up	−0.05	0.05	0.33	−0.09, 0.12	0.82
MOE: negative expectation → GPE 3‐month FU	−0.07	0.03	0.02	−0.14, −0.01	0.04
IE: pain baseline → negative expectation → GPE 3‐month FU	−0.004	NA	NA	−0.03, 0.007	0.42
*No mediation effect*					

Abbreviations: BCI, bootstrapped confidence interval; FU, follow‐up; GPE, global perceived effect; NA, not applicable; SE, standard error; SETS, Stanford Treatment Expectation Scale; SMT, spinal manipulative therapy.

^a^ Unstandardized parameter estimates.

All psychosocial variables (kinesiophobia, pain catastrophizing, depression, anxiety and pain self‐efficacy) partially mediated the relationship between baseline clinical back pain intensity and disability (6‐week and 3‐month follow‐up) for patients with CLBP submitted to SMT intervention (Table 7). The proportion mediated ranged from 8% to 25%; the higher values were observed for pain‐related catastrophizing and pain self‐efficacy (Table 7). Pain catastrophizing partially mediated the relationship between baseline pain intensity and pain intensity assessed at the 3‐month follow‐up. In addition, depression also partially mediated the relationship between baseline back pain intensity and back pain intensity assessed after the first session of SMT. The proportion mediated was 8% for both mediators on pain intensity (Table 7).

**TABLE 7:** Regression estimates of the mediation analysis, including baseline kinesiophobia (TSK), pain catastrophizing (PCS), depression (PHQ‐9), anxiety (GAD‐7) and pain self‐efficacy (PSEQ) as mediators and global perceived effect of improvement and disability outcomes as dependent variables (*n* = 264).

IVME = Independent Variable Mediator Effect (a path)	Estimate^a^	SE	*p*	95% CI	BCI *p*
MOE = Mediator‐Outcome Effect (b path)					
DE = Direct Effect (c path)					
IE = Indirect Effect (c' path)					
**Baseline kinesiophobia as mediator and baseline pain as independent variable**					
IVME: pain baseline → kinesiophobia	0.35	0.19	0.06	−0.01, 0.73	0.06
DE: pain baseline → pain immediately post‐SMT	0.52	0.05	0.001	0.41, 0.62	0.001
MOE: kinesiophobia → pain immediately post‐SMT	0.02	0.01	0.17	−0.01, 0.06	0.18
IE: pain baseline → kinesiophobia → pain immediately post‐SMT	0.008	NA	NA	−0.002, 0.03	0.13
*No mediation effect (only DE)*					
DE: pain baseline → pain 6‐week FU	0.01	0.02	0.50	0.36, 0.58	0.001
MOE: kinesiophobia → pain 6‐week FU	0.47	0.06	0.001	−0.02, 0.05	0.54
IE: pain baseline → kinesiophobia → pain 6‐week FU	0.004	NA	NA	−0.007, 0.03	0.30
*No mediation effect (only DE)*					
DE: pain baseline → pain 3‐month FU	0.42	0.06	0.001	0.26, 0.47	0.001
MOE: kinesiophobia → pain 3‐month FU	0.04	0.02	0.05	−0.004, 0.21	0.06
IE: pain baseline → kinesiophobia → pain 3‐month FU	0.01	NA	NA	0.000, 0.4	0.06
*No mediation effect (only DE)*					
DE: Pain baseline → disability 6‐week FU	1.04	0.31	0.001	0.41, 1.6	0.001
MOE: kinesiophobia → disability 6‐week FU	0.37	0.09	0.001	0.11, 0.33	0.001
IE: Pain baseline → kinesiophobia → Disability 6‐week FU	0.13	NA	NA	0.007, 0.34	0.04
*Partial mediation —* Proportion mediated	11% (95% CI: 0.06, 0.18)				
DE: pain baseline → disability 3‐month FU	1.03	0.32	0.001	0.69, 1.96	0.001
MOE: kinesiophobia → disability 3‐month FU	0.53	0.10	0.001	0.19, 0.39	0.001
IE: pain baseline → kinesiophobia → disability 3‐month FU	0.19	NA	NA	0.002, 0.43	0.04
*Partial mediation —* Proportion mediated	16% (95% CI: 0.10, 0.23)				
DE: pain baseline → GPE 6‐week FU	0.01	0.04	0.75	−0.09, 0.12	0.7
MOE: kinesiophobia → GPE 6‐week FU	−0.006	0.01	0.71	−0.13, 0.09	0.68
IE: pain baseline → kinesiophobia → GPE 6‐week FU	−0.002	NA	NA	−0.02, 0.006	0.48
*No mediation effect*					
DE: pain baseline → GPE 3‐month follow‐up	−0.06	0.05	0.28	−0.17, 0.05	0.31
MOE: kinesiophobia → GPE 3‐month FU	0.004	0.01	0.81	−0.09, 0.12	0.78
IE: pain baseline → kinesiophobia → GPE 3‐month FU	0.001	NA	NA	−0.009, 0.12	0.7
*No mediation effect*					
**Baseline catastrophizing as mediator and baseline pain as independent variable**					
IVME: pain baseline → catastrophizing	1.03	0.30	0.001	0.08, 0.32	0.002
DE: pain baseline → pain immediately post‐SMT	0.51	0.06	0.001	,37, 0.58	0.001
MOE: catastrophizing → pain immediately post‐SMT	0.01	0.01	0.24	−0.04, 0.18	0.27
IE: pain baseline → catastrophizing → pain immediately post‐SMT	0.01	NA	NA	−0.007, 0.04	0.20
*No mediation effect (only DE)*					
DE: pain baseline → pain 6‐week FU	0.47	0.06	0.001	0.32, 0.53	0.001
MOE: catastrophizing → pain 6‐week FU	0.01	0.01	0.24	−0.04, 0.18	0.24
IE: pain baseline → catastrophizing → pain 6‐week FU	0.015	NA	NA	−0.007, 0.05	0.16
*No mediation effect (only DE)*					
DE: pain baseline → pain 3‐month FU	0.39	0.06	0.001	0.08, 0.32	0.002
MOE: catastrophizing → pain 3‐month FU	0.04	0.01	0.005	0.05, 0.27	0.006
IE: pain baseline → catastrophizing → pain 3‐month FU	0.04	NA	NA	0.01, 0.09	0.003
*Partial mediation —* Proportion mediated	8% (95% CI: 0.04, 0.21)				
DE: pain baseline → disability 6‐week FU	0.94	0.32	0.003	0.05, 0.29	0.006
MOE: catastrophizing → disability 6‐week FU	0.23	0.06	0.001	0.08, 0.33	0.002
IE: pain baseline → catastrophizing → disability 6‐week FU	0.24	NA	NA	0.08, 0.49	0.002
*Partial mediation —* Proportion mediated	20% (95% CI: 0.13, 0.28)				
DE: pain baseline → disability 3‐month follow‐up	1.2	0.33	< 0.001	0.57, 1.8	0.001
MOE: catastrophizing → disability 3‐month follow‐up	0.28	0.06	< 0.001	0.12, 0.36	0.001
IE: pain baseline → catastrophizing → disability 3‐month FU	0.29	NA	NA	0.11, 0.56	0.001
*Partial mediation —* Proportion mediated	19% (95% CI: 0.14, 0.27)				
DE: pain baseline → GPE 6‐week FU	0.02	0.05	0.66	−0.07, 0.12	0.68
MOE: catastrophizing → GPE 6‐week FU	−0.08	0.01	0.42	−0.35, 0.008	0.30
IE: pain baseline → catastrophizing → GPE 6‐week FU	−0.008	NA	NA	−0.03, 0.008	0.3
*No mediation effect*					
DE: pain baseline → GPE 3‐month follow‐up	−0.05	0.05	0.36	−0.16, 0.05	0.36
MOE: catastrophizing → GPE 3‐month follow‐up	−0.005	0.01	−0.49	−0.14, 0.09	0.63
IE: pain baseline → catastrophizing → GPE 3‐month FU	−0.006	NA	NA	−0.03, 0.01	0.53
*No mediation effect*					
**Baseline depression as mediator and baseline pain as independent variable**					
IVME: pain baseline → depression	0.70	0.16	< 0.001	0.37, 1.0	0.007
DE: pain baseline → pain immediately post‐SMT	0.48	0.96	< 0.001	0.37, 0.59	< 0.001
MOE: depression → pain immediately post‐SMT	0.05	0.02	0.008	0.03, 0.25	0.01
IE: pain baseline → depression → pain immediately post‐SMT	0.04	NA	NA	0.001, 0.08	0.006
*Partial mediation —* Proportion mediated	8% (95% CI: 0.03, 0.18)				
DE: pain baseline → pain 6‐week FU	0.48	0.05	< 0.001	0.35, 0.6	< 0.001
MOE: depression → pain 6‐week FU	0.002	0.02	0.94	−0.1, 0.12	0.96
IE: pain baseline → depression → pain 6‐week FU	0.44	NA	NA	−0.03, 0.03	0.97
*No mediation effect (only DE)*					
DE: pain baseline → pain 3‐month FU	0.45	0.06	< 0.001	0.33, 0.57	< 0.001
MOE: depression → pain 3‐month FU	−0.03	0.02	0.19	−0.19, 0.03	0.20
IE: pain baseline → depression → pain 3‐month FU	−0.02	NA	NA	−0.06, 0.008	0.14
*No mediation effect (only DE)*					
DE: pain baseline → disability 6‐week FU	0.93	0.33	0.004	0.27, 1.61	0.006
MOE: depression → disability 6‐week FU	0.34	0.12	0.003	0.04, 0.30	0.007
IE: pain baseline → depression → disability 6‐week FU	0.17	NA	NA	0.06, 0.50	0.007
*Partial mediation —* Proportion mediated	15% (95% CI: 0.08, 0.21)				
DE: pain baseline → disability 3‐month follow‐up	1.28	0.34	< 0.001	0.64, 1.9	< 0.001
MOE: depression → disability 3‐month FU	0.30	0.12	0.01	0.05, 0.56	0.02
IE: pain baseline → depression → disability 3‐month FU	0.21	NA	NA	0.03, 0.47	0.02
*Partial mediation —* Proportion mediated	14% (95% CI: 0.08, 0.20)				
DE: pain baseline → GPE 6‐week FU	0.02	0.05	0.67	−0.07, 0.12	0.71
MOE: depression → GPE 6‐week FU	−0.01	0.02	0.56	−0.15, 0.08	0.53
IE: pain baseline → depression → GPE 6‐week FU	−0.007	NA	NA	−0.04, 0.01	0.46
*No mediation effect*					
DE: pain baseline → GPE 3‐month follow‐up	−0.04	0.05	0.34	−0.16, 0.06	0.35
MOE depression → GPE 3‐month FU	−0.005	0.02	0.80	−0.15, 0.11	0.76
IE: pain baseline → depression → GPE 3‐month FU	−0.003	NA	NA	−0.04, 0.02	0.75
*No mediation effect*					
**Baseline Anxiety as mediator and baseline pain as independent variable**					
IVME: pain baseline → anxiety	0.42	0.15	0.006	0.05, 0.27	0.003
DE: pain baseline → pain immediately post‐SMT	0.51	0.06	< 0.001	0.39, 0.62	< 0.001
MOE: anxiety → pain immediately post‐SMT	0.03	0.02	0.13	−0.3, 0.19	0.15
IE: pain baseline → anxiety → pain immediately post‐SMT	0.01	NA	NA	−0.003, 0.04	0.10
*No mediation effect (only DE)*					
DE: pain baseline → pain 6‐week FU	0.47	0.06	< 0.001	0.36, 0.57	< 0.001
MOE: anxiety → pain 6‐week FU	0.01	0.02	0.50	−0.7, 0.15	0.54
IE: pain baseline → anxiety → pain 6‐week FU	0.007	NA	NA	−0.01, 0.03	0.42
*No mediation effect (only DE)*					
DE: pain baseline → pain 3‐month FU	0.43	0.06	< 0.001	0.31, 0.55	< 0.001
MOE: anxiety → pain 3‐month FU	−0.002	0.03	0.95	−0.11, 0.11	0.94
IE: pain baseline → anxiety → pain 3‐month FU	−0.001	NA	NA	−0.02, 0.02	0.93
*No mediation effect (only DE)*					
DE: pain baseline → disability 6‐week FU	1.03	0.32	0.001	0.39, 1.67	0.001
MOE: anxiety → disability 6‐week FU	0.34	0.12	0.007	0.004, 0.28	0.01
IE: pain baseline → anxiety → disability 6‐week FU	0.14	NA	NA	0.04, 0.33	0.006
*Partial mediation —* Proportion mediated	12% (95% CI: 0.06, 0.19)				
DE: pain baseline → disability 3‐month follow‐up	1.32	0.34	< 0.001	0.68, 1.95	< 0.001
MOE: anxiety → disability 3‐month follow‐up	0.42	0.13	0.001	0.15, 0.69	0.003
IE: pain baseline → anxiety → disability 3‐month FU	0.18	NA	NA	0.05, 0.41	0.002
*Partial mediation —* Proportion mediated	12% (95% CI: 0.07, 0.17)				
DE: pain baseline → GPE 6‐week FU	0.02	0.05	0.67	−0.08, 0.12	0.72
MOE: anxiety → GPE 6‐week FU	−0.01	0.02	0.41	−0.05, 0.02	0.37
IE: pain baseline → anxiety → GPE 6‐week FU	−0.007	NA	NA	−0.03, 0.007	0.29
*No mediation effect*					
DE: pain baseline → GPE 3‐month follow‐up	−0.05	0.05	0.32	−0.16, 0.05	0.33
MOE: anxiety → GPE 3‐month follow‐up	−0.003	0.02	0.87	−0.04, 0.04	0.87
IE: pain baseline → anxiety → GPE 3‐month FU	−0.001	NA	NA	−0.02, 0.02	0.85
*No mediation effect*					
**Baseline pain self‐efficacy as mediator and baseline pain as independent variable**					
IVME: pain baseline → self‐efficacy	−0.93	0.29	0.001	−0.31, −0.06	0.001
DE: pain baseline → pain immediately post‐SMT	0.53	0.06	< 0.001	0.39, 0.59	0.001
MOE: self‐efficacy → pain immediately post‐SMT	0.000	0.01	−0.008	−0.12, 0.11	0.96
IE: pain baseline → self‐efficacy → pain immediately post‐SMT	0.000	NA	NA	−0.02, 0.02	0.96
*No mediation effect (only IVME and DE)*					
DE: pain baseline → pain 6‐week FU	0.48	0.06	< 0.001	0.33, 0.54	0.001
DE: self‐efficacy → pain 6‐week FU	−0.003	0.01	0.78	−0.13, 0.11	0.81
IE: pain baseline → self‐efficacy → pain 6‐week FU	0.003	NA	NA	−0.02, 0.03	0.73
*No mediation effect (only DE)*					
DE: pain baseline → pain 3‐month FU	0.42	0.06	< 0.001	0.27, 0.48	0.001
MOE: self‐efficacy → pain 3‐month FU	−0.007	0.01	0.62	−0.14, 0.09	0.63
IE: pain baseline → self‐efficacy → pain 3‐month FU	0.006	NA	NA	−0.02, 0.03	0.54
*No mediation effect (only DE)*					
DE: pain baseline → disability 6‐week FU	0.88	0.31	0.005	0.27, 1.47	0.004
MOE: self‐efficacy → disability 6‐week FU	−0.31	0.06	< 0.001	−0.44, −0.17	< 0.001
IE: pain baseline → pain self‐efficacy → disability 6‐week FU	0.29	NA	NA	0.11, 0.55	0.001
*Partial mediation —* Proportion mediated	25% (95% CI: 0.16, 0.33)				
DE: pain baseline → disability 3‐month follow‐up	1.21	0.33	< 0.001	0.59, 1.85	< 0.001
MOE: self‐efficacy → disability 3‐month FU	−0.31	0.07	< 0.001	−0.46, −0.15	< 0.001
IE: pain baseline → self‐efficacy → disability 3‐month FU	0.29	NA	NA	0.09, 0.57	0.001
*Partial mediation*	19% (95% CI: 0.14, 0.26)				
DE: pain baseline → GPE 6‐week FU	0.02	0.05	0.64	−0.08, 0.13	0.68
MOE: self‐efficacy → GPE 6‐week FU	0.01	0.01	0.31	−0.006, 0.03	0.21
IE: pain baseline → self‐efficacy → GPE 6‐week FU	−0.01	NA	NA	−0.03, 0.004	0.14
*No mediation effect*					
Direct effect: pain baseline → GPE 3‐month follow‐up	−0.06	0.05	0.31	−0.16, 0.06	0.33
MOE: self‐efficacy → GPE 3‐month FU	0.000	0.01	0.98	−0.01,0.02	0.94
Indirect effects: pain baseline → self‐efficacy → GPE 3‐month FU	0.000	NA	NA	−0.02, 0.02	0.96
*No mediation effect*					

Abbreviations: BCI, bootstrapped confidence interval; FU, follow‐up; GAD‐7, Generalized Anxiety Disorder Scale; GPE, global perceived effect; NA, not applicable; PCS, Pain Catastrophizing Scale; PHQ‐9, Patient Health Questionnaire; SE, standard error; SMT, spinal manipulative therapy; TSK, Tampa Scale for Kinesiophobia.

^a^ Unstandardized parameter estimates.

## 4 Discussion

This study aimed to investigate whether a hidden conditioning paradigm with positive expectations about SMT would result in greater reductions in clinical back pain intensity and/or higher global perceptions of effect for patients with CLBP, when compared with positive and neutral expectations alone. Furthermore, our findings showed that for SMT, as the treatment, there was no addictive effect of conditioning with positive expectation for any of the secondary outcomes when compared to positive or neutral expectations alone.

A pivotal aspect to interpret our results is the fidelity of the conditioning paradigm. Our results showed between‐group differences for mean experimental pain sensitivity in C‐PEG compared to the other two groups during the conditioning phase of the study, suggesting that participants perceived a decrease in the intensity of the heat‐painful stimulus applied after the first SMT session. However, no difference was observed between groups for clinical back pain intensity after the conditioning test (after SMT) in the first session of treatment, or for any other follow‐up sessions. This suggests that the patients in the C‐PEG did not generalize the association learned—between the SMT delivered, and the decrease in the pain intensity following the noxious heat stimulus during the conditioning—to the pain intensity related to back pain. The literature recognizes that pain conditioning is context‐dependent (Vlaeyen 2015), and our results confirm this assertion by demonstrating that participants may be strictly conditioned to heat pain.

Kong et al. (2018) studied knee osteoarthritis patients, comparing standard acupuncture with verbal suggestion and hidden conditioning, acupuncture alone, and a control group. Differing from our results, the results from Kong et al. (2018) showed greater pain intensity reduction favouring the group submitted to verbal suggestion and the conditioning procedure. In the former study, for the groups of comparison, no verbal suggestion was delivered, and the expectations suggestion was reinforced during the study, which may explain the differing findings. The absence of a group submitted to verbal suggestion alone in Kong et al.'s study prevents clarifying whether the effect observed was due to the conditioning, verbal suggestion or the combined effect.

Furthermore, a reduction in the mean positive expectations score was noted following participants' exposure to the videos, suggesting an inversion of the initially hypothesized effect for the positive expectation groups, especially regarding the SETS score. This decrease in the mean positive expectation score, with no between‐group difference in total expectation scores assessed after watching the videos, may suggest that the videos' content failed to modulate expectations. However, a recalibration of patients' expectations, fostering more realistic expectations, also may account for this finding, as the verbal positive suggestion was not imperative, suggesting a probability and not a certainty of the SMT efficacy (‘it is likely you will perceive an improvement in your back pain after manual therapy’). We adopted this speech to avoid an unethical practice. Additionally, both the C‐PEG and PEG groups rated treatment effectiveness (Q1 from the SETS) higher than the NEUG group, suggesting that the verbal suggestion affects positive and neutral groups differently, which can be considered evidence of the effectiveness of expectation manipulation. It is noteworthy that participants already had high treatment expectations at the baseline with scores over 84% on all scales, which is consistent with previous chronic pain studies (Geurts et al. 2017). This ceiling effect may have also constrained changes in expectation score pre‐ and post‐videos.

In addition, a previous study (Campanha et al. 2026) used a similar paradigm to investigate the effect of positive, neutral, and negative suggestions associated with SMT in CLBP and showed a significant clinical low back pain intensity reduction for the groups submitted to positive and neutral expectations compared to the group submitted to negative expectations. One can argue that the absence of a negative suggestion group or that the groups perceived positive and neutral expectations similarly may also be a reasonable explanation for the findings from this study.

An interaction between the various effects introduced in this study may account for our findings, resulting in subadditivity rather than additivity, where the combined treatment effect is less than the aggregate of individual active and contextual effects (Coleshill et al. 2018). Consequently, the contextual effects elicited through conditioning and/or verbal suggestion could have interacted with the active effects of SMT, thereby contributing to subtract the overall treatment outcome. A previous RCT that administered a similar SMT protocol for CLBP reported a mean within‐group change immediately after 4 weeks of SMT of 3.93 units (95% CI: 2.96, 4.89) (Tavares et al. 2023). Our findings are comparable but smaller than the results previously described and may support the subadditivity effect (Boussageon et al. 2022) between the effect promoted by the positive/neutral expectations and the effect of SMT.

For the mediation analysis investigating the effect of expectation and psychosocial variables in the relationship between baseline clinical back pain intensity and the outcomes of the study, our results showed that in the context of SMT, only positive expectation (not negative expectation) partially mediated the relationship between baseline clinical back pain intensity and disability and fully explained the relationship between baseline pain intensity and GPE. All psychosocial variables (kinesiophobia, catastrophizing, depression, anxiety and self‐efficacy) partially mediated the relationship between baseline clinical back pain intensity and disability. Only pain catastrophizing and depression partially explained the relationship between baseline clinical back pain intensity and clinical back pain intensity after SMT intervention. Such results showed that positive expectations and psychosocial factors are mediators of the effects of SMT on pain intensity, global perceived effect, and disability for patients with CLBP.

The full mediation observed in our study showed that the relationship between pain intensity and the GPE of improvement is completely dependent on positive expectation. Such findings suggest that improvements in GPE scores relied on positive expectations for patients with CLBP receiving SMT. In a previous mixed methods study, 65% of the patients with neck pain reported the treatment process as a contributing factor explaining GPE of improvement (Evans et al. 2014).

Additionally, the mediation analyses suggest that psychosocial variables are partially responsible for the improvement in disability. Thus, when SMT is applied with positive/neutral messaging (which is typically done clinically), it can impact disability levels partially through the influence of psychosocial variables. Previous studies confirm that psychosocial variables are predictors of disability (Kong et al. 2018) or mediate the relationship between disability and pain (Lee et al. 2021). Although the mediation findings must be interpreted with caution as there were no between‐group differences, they reinforce the influence of psychosocial factors on the effects of SMT for the management of CLBP.

This study has some strengths: (1) rigorous design, (2) high control of treatments delivered, (3) greater follow‐up period, (4) inclusion of patients and not healthy participants, and (5) large sample size. This study also has some limitations. (1) Although the study recruited a substantial number of participants (*n* = 264), the sample may not be fully representative of all populations with CLBP, particularly due to social vulnerability. In developing countries, pain management assistance is limited, and the study provided specialized free treatment, which may have screened a sample with tendentially higher expectations for improvement, or maybe higher expectations could be a characteristic of chronic pain patients. (2) Our control group was submitted to a neutral verbal suggestion, and the absence of a group without any type of intervention (natural history of disease) or SMT alone may limit the ability to distinguish the specific effect related to SMT from the effects caused by verbal suggestion and/or conditioning. The reason to include a neutral verbal suggestion group was to mimic clinical practice, since it is not reasonable to assume no communication about the treatment between patients and providers. (3) Another important aspect is the absence of a group submitted to negative verbal suggestions, since previous studies showed differences (Campanha et al. 2026), and (4) we cannot disregard a possible failure in expectation manipulation since a decrease in expectation was observed after participants watched the videos, for one of the measures used in the study. Nevertheless, the results may simply show that individuals who already have high expectations are unlikely to alter their views even when presented with extra information.

The results of this study have practical implications. Our findings showed that the approaches combining conditioning with positive verbal suggestions, and positive and neutral expectations alone yielded similar changes in back pain outcomes. Such findings suggest that the patients did not generalize the association learned between the SMT delivered and the decrease in pain intensity following the noxious stimulus during the conditioning to the pain intensity related to back pain. However, it's important to note that our participants brought high treatment expectations into the therapeutic setting, which may have created a ceiling effect for this variable. Independent of treatment assignment, positive expectation, along with psychosocial factors, partially mediated the association between baseline clinical back pain intensity and disability. Collectively, these findings indicate that positive expectations and psychosocial factors might play an important mediating role for a variety of outcomes in clinical practice for patients with CLBP receiving SMT.

## Conflicts of Interest

The authors declare no conflicts of interest.

## Supporting information


**File S1:** Study protocol.

**File S2:** The video content.

**File S3:** Directed acyclic graphs representation.
**Figure S1:** The path diagrams illustrate the directed acyclic graphs (DAGs) for the mediation analysis adopted in the study. The mediation variable is highlighted in the grey rectangle. The outcomes (black rectangle) were assessed at 1‐month and 3‐month follow‐ups. The DE is the effect of the independent variable on the outcome (dependent variable), excluding the influence of the mediator (letter c'). The MOE is the direct effect of the mediator on the outcome (letter b), the IVME is the effect observed between the independent variable and the mediator (letter a), and IE is the effect of the independent variable on the outcome (dependent variable) mediated by the positive/negative expectation or psychosocial variables (obtained by multiplying a*b).

**File S4:** AGReMA‐SF checklist.

**File S5:** CONSORT checklist.

## Data Availability

The data that support the findings of this study are not openly available due to ethical reasons but can be made available from the corresponding author upon reasonable request. Access to the data will be restricted to ensure that data are only made available to researchers for ethically approved research projects, on the understanding that confidentiality will be maintained.
