# Supplementary material for: Effects of Hidden Conditioning and Positive Expectations on Pain Intensity Following Spinal Manipulative Therapy: A Three‐Arm Randomized Controlled Trial
Source: Eur J Pain. 2026 Oct 12;30(9):e70414. doi: 10.1002/ejp.70414 (PMC13656883; doi:10.1002/ejp.70414)
Supplement: Supplementary file 1 [file EJP-30-0-s001.pdf]

BMJ Open

Investigating the hypoalgesic effects of spinal manipulative therapy using hidden pain conditioning and positive expectation in patients with chronic low back pain: protocol for a randomised controlled trial

Helen Cristina Nogueira Carrer,[1] Thamiris Costa de Lima,[1] Steven Z George,[2] Felipe José Jandre dos Reis,[3] Deise Lilian Costa Dias,[1] Bianca Emanuelli Saes Campanha,[4] Thaís Cristina Chaves [1,5]

To cite: Nogueira Carrer HC, Lima TC, George SZ, *et al*. Investigating the hypoalgesic effects of spinal manipulative therapy using hidden pain conditioning and positive expectation in patients with chronic low back pain: protocol for a randomised controlled trial. *BMJ Open* 2023;**13**:e066199. doi:10.1136/bmjopen-2022-066199

► Prepublication history and additional supplemental material for this paper are available online. To view these files, please visit the journal online (http://dx.doi.org/10.1136/bmjopen-2022-066199).

Received 04 July 2022
Accepted 22 March 2023

Check for updates

© Author(s) (or their employer(s)) 2023. Re-use permitted under CC BY-NC. No commercial re-use. See rights and permissions. Published by BMJ.

For numbered affiliations see end of article.

Correspondence to
Dr Thaís Cristina Chaves; thaischaves@ufscar.br

ABSTRACT

Introduction Placebo effects are responses capable of modulating pain and influencing treatment response. Two mechanisms are commonly related to placebo effects: expectations and conditioning. However, the research in this field is focused on laboratory studies with healthy participants. This study aims to identify whether a conditioning procedure with positive induced expectations about spinal manipulative therapy (SMT) will result in greater hypoalgesic effects in adults with chronic low back pain (CLBP) in a clinical trial design.

Methods and analysis This trial will enrol 264 patients with non-specific CLBP, aged 18–60 years. Patients will undergo a calibration test to determine the thermal pain threshold for the hidden pain conditioning procedure. Afterward, they will be randomised to one of the three groups: hidden pain conditioning with positive induced expectations—group one (G1); positive expectations—group two (G2) and neutral expectations—group three (G3). Patients will receive instructions to manipulate the expectations. The pretreatment heat pain test will be performed before the SMT and after the intervention patients will undergo again the heat pain intensity test. However, only patients in G1 will receive hidden pain conditioning to reinforce the association between SMT and pain intensity reduction. All patients will undergo five sessions of SMT. The outcomes will be assessed immediately after the last session and at the 6 weeks and 3-month follow-ups. All statistical analyses will be conducted following intention-to-treat principles, and the treatment effects will be determined with linear mixed models.

Ethics and dissemination The Federal University of São Carlos approved this research (Process n° 52359521.1.0000.5504). All participants will give written informed consent. Dissemination of the results will include publications in peer-reviewed journals and presentations at conferences. If positive expectations and classical conditioning improve outcomes, it may support the administration of such intervention.

Trial registration number NCT05202704.

STRENGTHS AND LIMITATIONS OF THIS STUDY

⇒ The main strength of this three-arm randomised controlled trial is to investigate the effect of positive expectations about spinal manipulative therapy associated with pain classical conditioning on pain intensity and global perceived effect of improvement when compared with two additional groups: one submitted to positive expectations alone and another submitted to neutral expectations about spinal manipulative therapy.

⇒ The large sample size considered and the interim assessment of outcomes (3-month follow-up) are additional new contributions of this clinical trial.

⇒ Patients will be submitted to a procedure in which intense nociceptive thermal stimulation will be administered for classical conditioning. This procedure could increase the likelihood of dropout, but they will be previously familiarised with the procedure which we expect will minimise the fear of the quantitative sensory testing.

⇒ We will only recruit chronic non-specific low back pain, which will mean our results are generalisable to the most commonly experienced type of low back pain (prevalence cited as high as 90%).

⇒ This study will be conducted in the public setting in the outpatient rehabilitation centre at the Federal University of São Carlos. A previous systematic, qualitative meta-summary and meta-synthesis reported the effect of the healthcare setting as a determinant for patient satisfaction with the treatment. Although we did not control for satisfaction in the current study, we recognise that healthcare setting is a contextual factor that could influence our results. On the other hand, the outpatient rehabilitation service where the study will be conducted is a facility with excellent features and equivalent (or superior) to private clinical environments.

BMJ Open: first published as 10.1136/bmjopen-2022-066199 on 12 April 2023. Downloaded from http://bmjopen.bmj.com/ on April 27, 2023 by guest. Protected by copyright.

INTRODUCTION

The point prevalence of low back pain (LBP) in 2017 was estimated to be about 7.5% of the global population, or around 577.0 million people.[1] In the Global Burden of Diseases study, LBP was the fourth cause of disability-adjusted life-years in the 25–49-year age group,[2] and most LBP is termed as non-specific, in which no specific pathoanatomic cause can be identified.[3 4] LBP localised below the costal margin and above the inferior gluteal folds, with or without leg pain, that persists for more than 3 months is defined as chronic low back pain (CLBP).[5 6] CLBP is commonly associated with psychosocial impacts and disability.[4]

For the treatment of LBP, guidelines recommend the use of non-pharmacological therapies such as spinal manipulative therapy (SMT).[6–8] A meta-analysis showed that SMT has similar effects to other recommended non-pharmacological therapies showing small clinical effects for pain relief and improved functioning.[9] Changes in patient outcomes have been attributed to confounders (eg, the natural history of the disease), statistical factors (eg, regression to the mean), specific treatment effects (eg, biological/active component) and non-specific or contextual factors, also recognised as the placebo effect.[9 10] Specific to SMT, Bialosky *et al*[11] have described theoretical models for how expectations and contextual factors influence pain-related responses to SMT. A complex interaction of factors related to the patient, the provider and the environment could lead to pain inhibition.[11–15]

A contextual or placebo effect can be defined as a genuine psychobiological event attributable to the overall therapeutic context.[16] The placebo effect can be a large contribution to the treatment response and it has been argued that enhancing this effect could improve the effectiveness of interventions commonly used in clinical practice.[17]

Two mechanisms commonly related to the placebo effect are expectations and classical conditioning.[18] Expectations can be defined as beliefs and predictions about future events or outcomes.[19] Expectations can be positively modulated using verbal suggestions with instructions about the benefits of an individual intervention.[20–22] Another mechanism underlying placebo effects involves classical conditioning.[16] For example, in the context of placebo hypoalgesia, classical conditioning is typically induced in the laboratory using a 'response conditioning' paradigm, where treatment cues (eg, cream or injection) are paired with surreptitious reductions in the intensity of painful stimuli.[23–25] The procedure has also been called hidden pain conditioning since the patient is unaware of the downgrading of the intensity of the stimulus in the test post-intervention. As a result, the patient is induced to learn a positive association between the treatment and the hypoalgesic effect.[25 26]

A systematic review assessed the pain-reducing effects of expectation, classical conditioning and imagery in patients with pain and found a medium to large effect for verbal suggestion and a medium to small effect for conditioning.[16] The authors clearly stated the lack of studies about conditioning in clinical trial designs. There are previous studies that investigated the effect of verbal suggestion associated with SMT.[21 27] Malfliet *et al*[16] showed a significant pain reduction after SMT in patients with chronic neck pain that received positive or neutral expectations about the SMT when compared with the negative expectation group. Additionally, no previous study compared the hypoalgesic effects associated with positive expectation and hidden pain conditioning procedure versus positive expectation alone in patients with CLBP, when submitted to SMT. A growing body of research suggests that adjusting multiple contextual factors may have a greater impact on the clinical outcomes of individuals with CLBP.[28]

Thus, the objective of this randomised controlled trial will be to investigate whether a hidden pain conditioning procedure associated with induced positive expectations about SMT will result in greater hypoalgesic effects when compared with positive or neutral expectations alone for patients with CLBP. Our primary hypothesis is that the group submitted to positive expectation+pain conditioning+SMT will have greater short-term pain intensity reduction, than the other treatment groups (positive expectation+SMT or neutral expectation+SMT). We also hypothesised that the patients submitted to positive expectations about the treatment will show greater pain reduction than the group who will be submitted to neutral expectations.

METHODS

Trial design

This study will be a randomised controlled trial with three parallel arms, following the recommendations of an extension of the Consolidated Standards of Reporting Trials (CONSORT).[29] The CONSORT flowchart is represented in figure 1. This protocol will follow the Standard Protocol Items: Recommendations for Interventional Trials (SPIRIT).[30] The trial was authorised by the ethics committee for research involving human subjects of the Federal University of São Carlos (Process N° 52359521.1.0000.5504). The study was registered prospectively on ClinicalTrials.gov.

Patient and public involvement

Participants in this study were not involved in the development of the study design or objectives. The research design and objectives were developed by the investigators of this study and underwent review by ad hoc assessors from the FAPESP (The São Paulo Research Foundation) that supports this study. However, we conducted a pilot study with 10 members of the community (service users from the Outpatient Clinic at the Federal University of São Carlos) at the beginning of 2022. The service users provided their comments on the questionnaires and procedures adopted in the current study. All the questionnaires used in the study were tested previously with

BMJ Open: first published as 10.1136/bmjopen-2022-066199 on 12 April 2023. Downloaded from http://bmjopen.bmj.com/ on April 27, 2023 by guest. Protected by copyright.

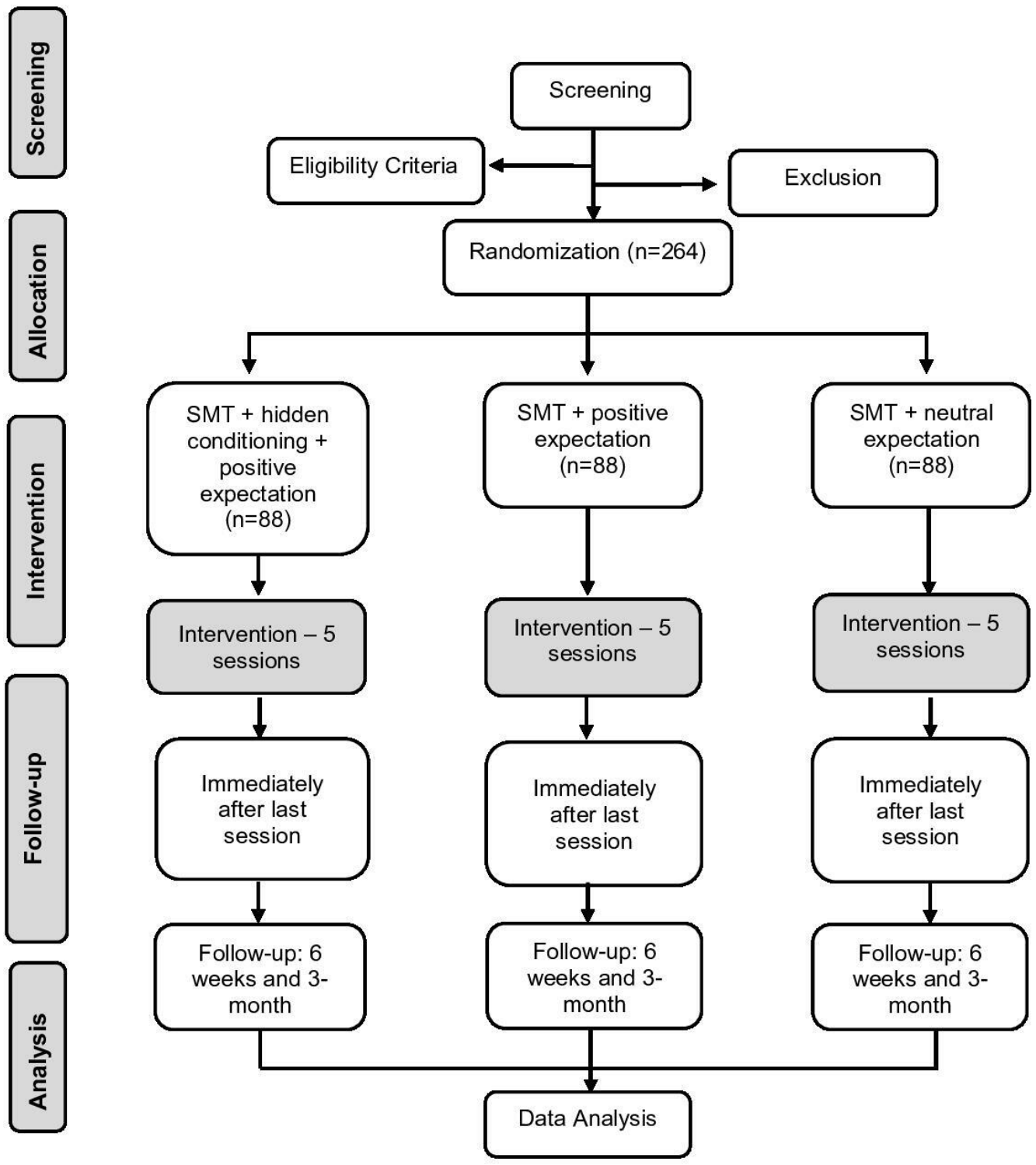

Figure 1 The Consolidated Standards of Reporting Trials (CONSORT) flowchart illustrates the randomisation process that will be employed in this clinical trial. SMT, Spinal Manipulative Therapy.

patients regarding comprehension. Furthermore, the results of the study will be disseminated to study participants and the wider public through social media and presentations organised by our research group. Finally, we will seek input from patients on the best ways to incorporate these treatments into clinical practice, if the results of the trial indicate the superiority of the positive expectations associated with pain conditioning and SMT.

Eligibility criteria and patients

A sample size of 264 patients with CLBP will participate in this study in a reference public outpatient rehabilitation service at the Federal University of São Carlos (UFSCar)—Brazil. This study will receive extensive exposure in local media and social networks. The study started recruiting in February 2022, and the estimated study completion date is in March 2024. The inclusion criteria for patients were as follows: (1) patients who report non-specific CLBP (in which the specific nociceptive source cannot be identified, confirmed by a medical assessment)[5] for at least 3 months duration[6]; (2) age ranged from 18 years to 60 years[31]; (3) baseline Pain Intensity Score ≥3 on a Numeric Pain Rating Scale (NPRS) (because of the measurement error >2 reported for the NPRS)[32]; (4) score greater than 14% on the Oswestry Disability Index (ODI)[33] (to surpass the smallest detectable change of the ODI total score); (5) patients able to speak and understand Portuguese well to fill out the questionnaires. Patients will be excluded if they met any of the following criteria: (1) Previous poor experiences with SMT through the application of a brief screening questionnaire[23]; (2) pregnancy[34]; (3) specific LBP disorders like radiculopathy or lumbar stenosis or chronic degenerative disorders, that is, uncontrolled cardiovascular, metabolic or systemic diseases, neurological or psychiatric diseases, and stroke sequelae[33]; (4) undergoing other therapeutic interventions for chronic pain and LBP (including surgeries) in the last 3 months[35]; (5) presence of contraindications to SMT[36]; (6) patients who reach the upper limit of 50°C, which is the maximum temperature provided by the Q-sense equipment described in the Medoc, Israel website.[37]

Patients will be instructed to not use pharmacological pain relief while participating in the trial, but they will be encouraged to report their use if unavoidable.

Randomisation and allocation

Once the patient has accepted the invitation to participate and signed the formal consent to participate, one physiotherapist researcher (post-doctorate) will run the assessment to determine eligibility. After that, patients will be submitted to the calibration procedure to determine the heat pain threshold for the heat pain assessments, since patients can be excluded from the study if they hit the maximum pain threshold available for the Medoc Q-sense equipment. Figure 1 shows the trial procedure according to the CONSORT flowchart, and table 1 illustrates the trial schedule. In the event of two consecutive absences from treatment sessions, patients will be contacted by telephone or in-person by blinded research personnel.

After the calibration test, patients will be randomly assigned to, following simple computerised randomisation procedures, one of the three treatment groups through the use of cards previously placed in opaque sealed envelopes: G1—hidden pain conditioning and positive expectation; G2—positive expectation; G3—the control group—neutral expectation. The researcher involved in the randomisation and allocation procedures will be not blinded to the allocation of patients to the treatment groups.

Another blinded researcher regarding the group allocation will run the baseline assessments and collect data outcomes in all endpoints.

Procedures

Initially, patients will watch a video to promote the verbal suggestion (positive expectation for G1 and G2 and neutral expectation for G3). Second, the patients will be submitted to pretreatment heat pain assessment. Following, patients will be submitted to the first session of SMT and, immediately after, patients will be submitted again to the heat pain assessment test. In this post-treatment test, just the patients from G1 will be submitted to the hidden conditioning (surreptitious downgrade of the heat pain intensity). During the tests (pretreatment and post-treatment), as a manipulation check, the researcher (not blinded to group allocation) will

BMJ Open: first published as 10.1136/bmjopen-2022-066199 on 12 April 2023. Downloaded from http://bmjopen.bmj.com/ on April 27, 2023 by guest. Protected by copyright.

Table 1 Recommended protocol items: schedule for enrolment, interventions and assessments

	Enrolment	Baseline (session 1)	Session 2	Session 3–5	Session 6	Post-intervention	Follow-ups (6 weeks and 3 months)
Time point	$-t_1$	t_1				t_2	
Enrolment:							
Eligibility screening	X						
Informed consent	X						
Randomisation			X_1				
Allocation			X_1				
Interventions:							
(Calibration for thermal pain threshold)		X					
(Videos to promote expectations)			X_2				
(Pretreatment test—heat pain)			X				
(Post-treatment heat pain test—with or without pain conditioning)			X_3				
(Spinal manipulative therapy)			◆———	————	———◆		
Assessments:							
(Treatment expectations)		X					
(Expectations of improvement)		X					
(PHQ-9)		X					
(GAD 7)		X					
(PCS)		X					
(PANAS)		X					
(TSK)		X					
(Pain intensity)		X	X*			X	X
(Heat pain threshold)		X					
(Heat pain intensity)			X†				
(GPE)			X*			X	X
(ODI)		X				X	X
(PSEQ)		X					

X1: Randomisation and allocation after the calibration test to determine the thermal pain threshold for the heat pain assessments
X2: Induction of expectations (positive or negative), followed by pretreatment heat pain test
X3: Hidden pain conditioning procedure will be performed only for G1.
The allocation will only be performed after the calibration procedure (session 2).
*Immediately after the hidden pain conditioning procedure for G1 (manipulation check to confirm if the conditioning was effective) and after the post-treatment heat pain assessmnet for G2 and G3
†The heat pain intensity will be obtained before and after the manipulative therapy in the second session.
GAD-7, General Anxiety Disorder-7; GPE, Global Perceived Effect; ODI, Oswestry Disability Index; PANAS, Positive and Negative Affect Scale; PCS, Pain Catastrophizing Scale; PHQ-9, Patient Health Questionnaire-9; PSEQ, Pain Self-Efficacy Questionnaire; TSK, Tampa Scale for Kinesiophobia.

BMJ Open: first published as 10.1136/bmjopen-2022-066199 on 12 April 2023. Downloaded from http://bmjopen.bmj.com/ on April 27, 2023 by guest. Protected by copyright.

ask patients about pain intensity for each heat stimulus administered. After the pretreatment heat pain assessment procedure, a blinded researcher to group allocation will collect the primary and secondary outcomes (see below – Measures section) and the first session of SMT will be administered. Immediately after, a post-treatment heat pain assessment will be conducted in all participants, but only participans in G1 will be submitted to the hidden pain conditioning. Then, it will be followed by the collection of the primary and secondary outcomes again. In the following sessions, patients will be submitted to SMT once a week, for 5 weeks and after the fifth SMT session, the patients will be submitted to the post-treatment assessment to collect primary and secondary outcomes. Additionally, patients will be reassessed 3 months after randomisation.

Measures

Primary outcomes

Pain intensity

The NPRS will be used to assess pain intensity in this trial and consists of a sequence of numbers from 0 to 10, in which 0 represents 'no pain' and 10 represents 'worst pain imaginable'.[11] The Brazilian Portuguese version showed suitable reliability (intraclass correlation coefficient (ICC)=.92).[38] The minimal important change (MIC) value described for NPRS is 2 points.[39]

Participant ratings of Global Perceived Effect

The Global Perceived Effect (GPE) of improvement used for this trial is an 11-point scale that ranges from −5 ('very much worse') through 0 ('no change') to +5 ('completely recovered') and patients are asked: 'Compared to when this episode first started, how would you describe your low back pain these days?'. Higher score indicates higher perception of recovery from the condition.[38] The GPE Brazilian version showed acceptable reliability (ICC=.90–.99) and construct validity when compared with Roland-Morris Questionnaire (r=−.42).[38] MIC for GPE is 2 points.[40]

Secondary outcome measures

ODI

The ODI assesses disability related to CLBP. This instrument consists of 10 items, with six response options for each item. It ranges from 0 (absence or small amount of LBP and disability) to 5 points (extreme LBP and disability) and it has been validated in Brazilian Portuguese.[41] The score is calculated by adding items, with a maximum of 50 points that will be converted to 100 points by multiplying the final result by two. The ODI will be administered pretreatment, post-treatment and at the follow-ups. ODI Brazilian version showed suitable internal consistency (Cronbach's α=.87), reliability (ICC=.99) and acceptable construct validity when compared with Roland-Morris Questionnaire (r=.81).[41] Ten points are the MIC value for ODI.[42]

Baseline

Stanford Expectations of Treatment Scale

The Stanford Expectations of Treatment Scale (SETS) has six questions with seven response options for each item.[43] The positive expectation domain must encompass the sum of items one, three and five. The negative expectation domain sums up questions two, four and six. The scale will be administered pretreatment and soon after the hidden pain conditioning. The scale has been validated in Brazilian Portuguese, but the results are under review for publication. SETS showed suitable internal (Cronbach's α=.84–.87) and reliability (ICC=.72–.76).

LBP Improvement Expectation Scale

The LBP Expectation Scale assesses the patient's previous expectation that the LBP will improve. The participant is asked to respond using an 11-point scale, ranging from 0 to 10, where 0 represents 'no chance' and 10 represents 'very likely'.[44] The scale will be administered pretreatment and soon after the hidden pain conditioning procedure. The LBP Improvement Expectation Scale has been validated in Brazilian Portuguese, but the results are under review for publication. The LBP scale showed suitable reliability (ICC=.76).

Patient Health Questionnaire-9

The Patient Health Questionnaire-9 (PHQ-9) Brazilian version will be used to assess symptoms of depression.[45] Patients will be asked to rate nine questions on a 4-point scale over the 2 weeks before the administration of the questionnaire. Validity has been assessed against an independent structured mental health professional interview. The PHQ-9 Brazilian version showed suitable structural validity (Confirmatory Fit Index=.98).[46]

General Anxiety Disorder-7

The General Anxiety Disorder-7 (GAD-7) is a widely used diagnostic self-reported scale for anxiety disorder screening, diagnosis and severity assessment. Patients will be asked to rate their anxiety-related difficulties on a 4-point scale over the 2 weeks before the administration of the questionnaire. GAD-7 scores vary from 0 to 21, with higher GAD-7 scores being linked to more severe generalised anxiety. The measurement properties of the GAD-7 in Brazilian Portuguese[47] showed a one-dimensional structure and good internal consistency (Cronbach's α=.88).

Tampa Scale for Kinesiophobia

The Tampa Scale for Kinesiophobia (TSK) is a self-reported questionnaire that assesses the fear of movement. The scores for each of the 17 items ranged from 1 to 4 points. It is necessary to invert the scores of the questions 4, 8, 12 and 16 for the total score. The final score can range from 17 to 68 points, higher scores represent stronger beliefs about fear of movement.[48] The TSK Brazilian version showed acceptable internal consistency (Cronbach's α=.82) and reliability (ICC=.93).[48]

BMJ Open: first published as 10.1136/bmjopen-2022-066199 on 12 April 2023. Downloaded from http://bmjopen.bmj.com/ on April 27, 2023 by guest. Protected by copyright.

Pain Catastrophizing Scale

The Pain Catastrophizing Scale (PCS) Brazilian Portuguese is a self-administered questionnaire that consists of 13 items for the assessment of catastrophizing thoughts.[49] It is divided into three domains: helplessness, magnification and rumination. Each item is scored on a 5-point ordinal scale. The PCS total score ranges from 0 to 52 points, higher values denote greater pain catastrophizing. PCS Brazilian version showed suitable internal consistency (Cronbach's α=.86–.93) and construct validity when PCS was compared with Brief Pain Inventory (r=.48–.65).[49]

Positive and Negative Affect Scales

Positive and Negative Affect Scales will be used to assess the positive and negative affect constructs.[50] The scale is made up of 40 items, each factor made up of 20 adjectives that represent the subjects' moods and emotions. Each adjective is rated by the patients on a 5-point Likert scale. The Brazilian Portuguese[51] demonstrated acceptable measurement properties. The scale showed suitable internal consistency (Cronbach's α=.84) and structural validity showing infit and outfit values between 0.5 and 1.50.[51]

Pain Self-Efficacy Questionnaire

Study patients will be evaluated on self-efficacy related to chronic pain, which can be defined as an individual's confidence that he/she can successfully produce desirable results related to living with chronic pain. The Pain Self-Efficacy Questionnaire (PSEQ) has 10 items which are rated on a 7-point ordinal scale. It was cross-cultural adapted and validated to Brazilian Portuguese.[52] Previous research showed an effect on self-efficacy using a pain neuroscience education intervention based on metaphors compared with an intervention using cognitive-behavioural concepts.[53] PSEQ Brazilian version showed suitable internal consistency (Cronbach's α=.90) and construct validity when compared with Roland-Morris Questionnaire (r=−.58).[52]

Interventions

For all three groups, patients will undergo lumbar SMT in combination with verbal suggestions. The experimental group (G1) will also receive hidden pain conditioning. In table 2, the reader can find the description of the stages of the current study. Two follow-up sessions will also be scheduled 6 weeks and 3 months post-randomisation.

Calibration protocol to determine the heat pain threshold

All the procedures adopted in the current study for the calibration, to determine the heat pain threshold and the hidden pain conditioning protocol, were similar to the procedures described previously.[35] The researcher in charge of the hidden pain conditioning procedure is a physiotherapist and full post-doctoral researcher and was trained by the chief researcher for 60 hours to conduct and administrate the protocol. The chief researcher has experience in studies with quantitative sensory testing and pain conditioning protocols.[54 55] The calibration test will occur on session 1.

Patients will receive a series of progressive stimuli starting at 32°C. The stimuli will be increased by 1°C until reaching 50°C or the participant's maximum tolerance. Each stimulus will be held at each level for 5 s, including 2 s of stimulus ramp up and ramp down. The time interval between stimuli will be 30 s and subjects will rate their pain intensity using the Gracely Scale.[56] This scale has 20 increasing levels of pain perception and has demonstrated good levels of reliability.[56] Heat stimuli will be administered using the Q-sense device (Medoc) with a 3 cm × 3 cm thermode sensor.[57] Temperature values at which the

Table 2 The schedule of the interventions administered in the study

	Group 1	Group 2	Group 3
Session 1	Eligibility Assessment +calibration procedure - followed by Randomisation and Allocation<br>Baseline Assessment		
Session 2	► Positive expectation.<br>► Pre-conditioning test—for the assessment of the heat pain.<br>► First SMT session.<br>► Conditioning test—hidden pain conditioning—surreptitiously downgrade of the heat pain threshold (intense to weak).	► Positive expectation.<br>► Pretreatment test for the assessment of the heat pain.<br>► First SMT session.<br>► Post-treatment test for the assessment of the heat pain intensity.	► Neutral expectation.<br>► Pretreatment test for the assessment of the heat pain.<br>► First SMT session.<br>► Post-treatment test for the assessment of the heat pain intensity.
Sessions 3–5	► SMT (manipulations twice on each side and mobilisations for 1 min in each lumbar vertebra—total time: 10 min).	► SMT (manipulations twice on each side and mobilisations for 1 min in each lumbar vertebra—total time: 10 min).	► SMT (manipulations twice on each side and mobilisations for 1 min in each lumbar vertebra—total time: 10 min).
Session 6	► SMT+post-treatment assessment.	► SMT+post-treatment assessment.	► SMT+post-treatment assessment.

The spinal manipulative therapy (SMT) will be administered to the three groups of the study: Group 1—hidden pain conditioning with positive induced expectations; group 2—positive expectation group; group 3—neutral expectation group.

BMJ Open: first published as 10.1136/bmjopen-2022-066199 on 12 April 2023. Downloaded from http://bmjopen.bmj.com/ on April 27, 2023 by guest. Protected by copyright.

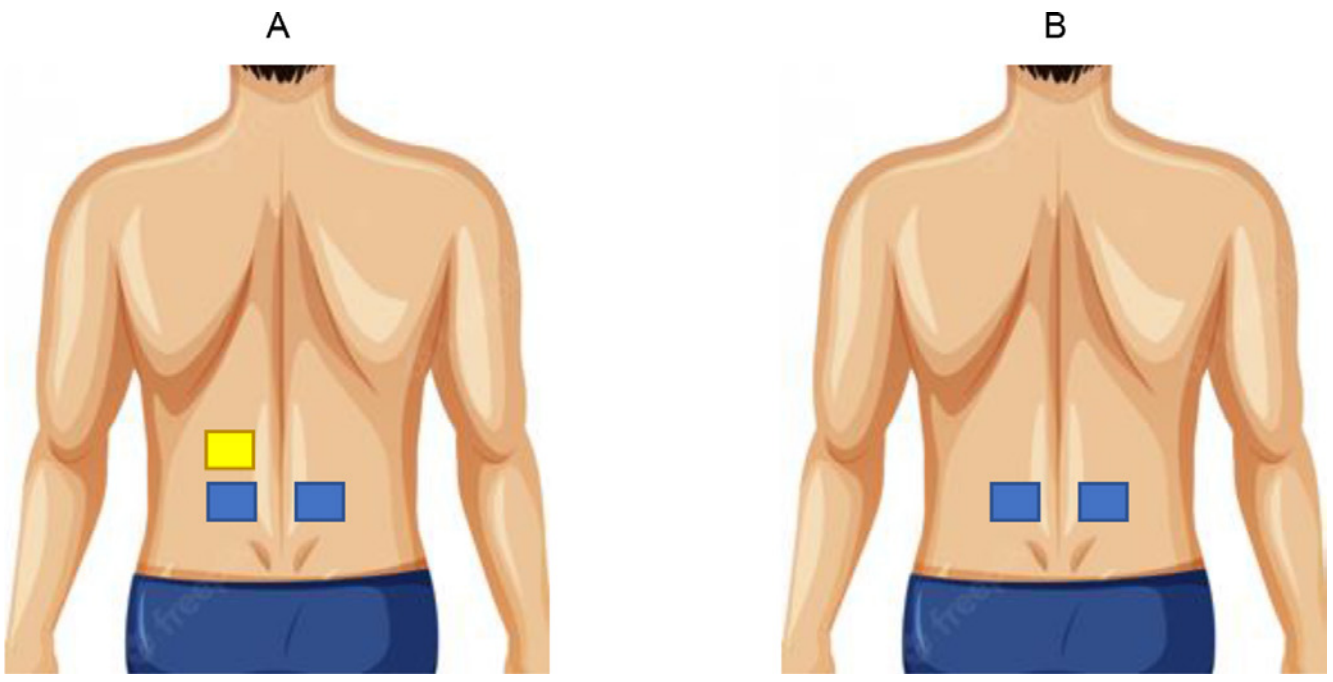

Figure 2 Sites in which the heat nociceptive stimulus will be administered during the heat pain test with and without hidden pain conditioning. (A) Sites considered during the pretreatment heat pain assessment (before spinal manipulative therapy). The severe nociceptive stimuli (SNS) will be administered over the blue rectangle sites and the random nociceptive stimuli (changing low/moderate and severe stimulus randomly) will be administered over the yellow rectangle sites. (B) Sites considered during the post-treatment heat pain intensity assessment (after the spinal manipulative therapy). The G1 (hidden pain conditioning+positive expectation) will receive heat stimulus over the areas of the blue rectangles (low/moderate nociceptive stimuli) and the other groups (G2—positive expectation and G3—neutral expectation) will receive SNS over the areas of the blue rectangles.

participant reports mild-to-moderate pain intensity (5–7 on the Gracely Scale) and severe pain intensity (14–15 on the Gracely Scale) will be recorded for each volunteer.[35] Heat pain thresholds will be obtained in two areas of the lumbar spine (blue rectangles) that will be delimited by the same size as the thermode (figure 2). Heat pain threshold values will be set for the two areas separately. If we observe a difference in heat pain threshold between both sides, the lower value will be considered for the hidden pain conditioning procedure. The region of the L3 vertebra will be used as a reference for delimitating the rectangles (figure 2).

Thermal pain assessment pre-SMT and post-SMT with and without hidden pain conditioning with noxious heat stimulus

The protocol will be divided into two steps: (1) Pretreatment heat pain assessment and (2) Post-treatment heat pain assessment with conditioning (G1) and without conditioning (G2 and G3).

In the pretreatment test, 14 nociceptive stimuli of severe intensity will be administered at random in the three locations (5 stimuli for each side of the low back) corresponding to the blue rectangles in the low back spine region (figure 2). As a means to investigate the accuracy of the procedure and improve the unpredictability of the test, another area in the low back (yellow rectangle—figure 3) will be submitted to four noxious thermal stimuli at random (low/moderate/severe stimulus). After each stimulus, the volunteers will be asked to rate their perceived pain intensity using the NPRS as a manipulation check.

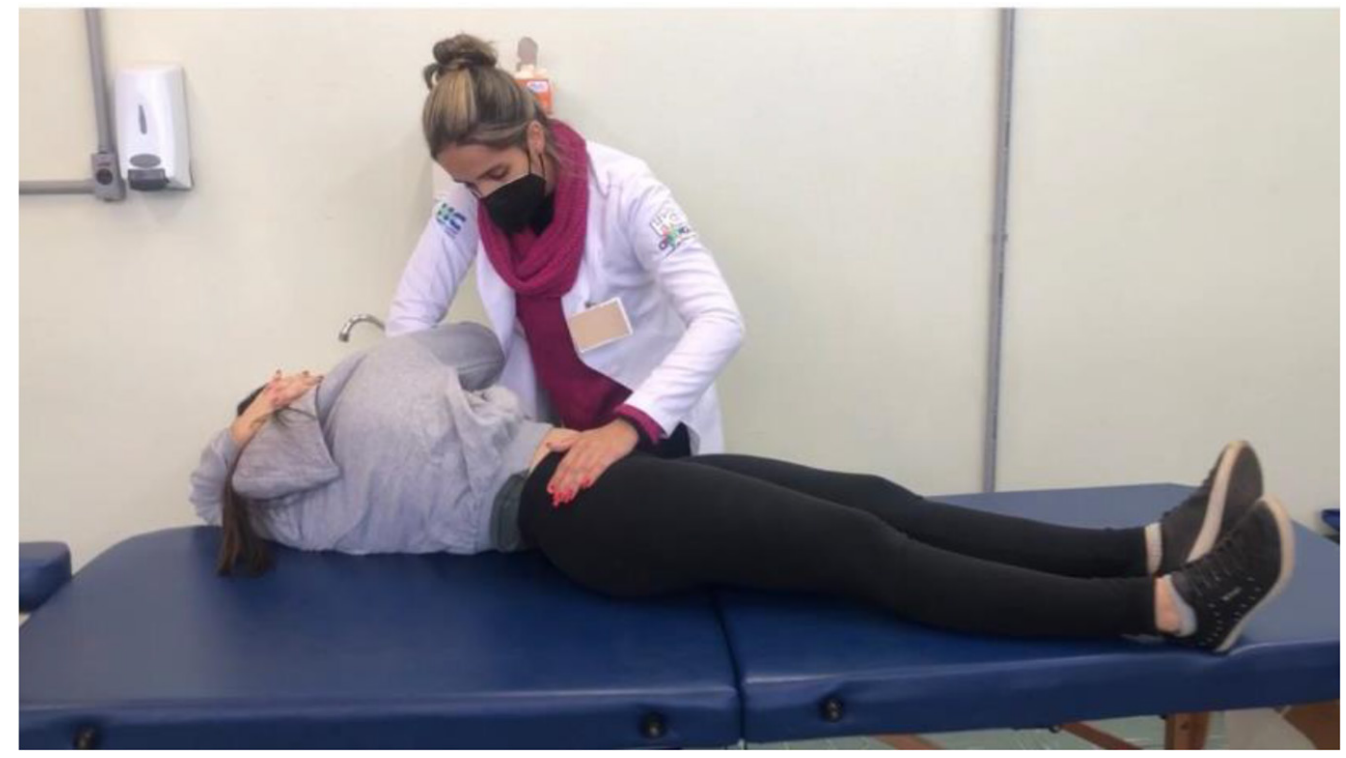

Figure 3 The spinal manipulative therapy technique that will be administered in the study. Global low-amplitude and high-speed manipulative manoeuvre administered in the sacroiliac joint.

In the sequence, patients will be submitted to the first SMT session and afterward post-treatment test (with and without conditioning) will be performed. A new sequence of stimuli consisting of five low/moderate intensity stimuli (downgrading the stimuli administered in the pretreatment test to promote the hidden pain conditioning) will be administered to the area marked with blue rectangles (figure 2) for the patients of the G1. Particularly for G2 and G3, stimuli with the same intensity (severe) as administered in the pretreatment test will be administered during the post-treatment. Each trial of nociceptive heat stimulation will be sustained for 5 s, including 2 s of ramp up and ramp down. The volunteer will be asked to rate the perceived heat pain intensity using NPRS for each site stimulated. This pretreatment and post-treatment heat pain assessment will be carried out by a researcher who will not be blinded to the group allocation since she will need to be aware that one of the groups (G1) will be given a lower threshold in the post-treatment heat pain assessment test. Pretreatment ad post-treatment thermal pain assessments will occur on session 2, after randomisation.

Verbal priming to promote positive or neutral expectations

Following allocation, each group will be given specific instructions (the patients will be invited to watch a video). The verbal priming will be administrated as a video to guarantee consistency among patients. The speech of the videos regarding positive and neutral expectations is available as an online supplemental file.

SMT

The SMT which will be administered in the current study has been widely used in the literature.[8 12 58–60] In this study, we consider SMT to represent any hands-on treatment of the spine, including both manipulation and mobilisation techniques applied to the spinal.[9] Mobilisations use low-grade velocity, small or large amplitude passive movement techniques within the patient's range of motion and control, whereas manipulation uses a high-velocity impulse (thrust) over a short amplitude at or near the end of the passive or physiological range of motion.[9]

BMJ Open: first published as 10.1136/bmjopen-2022-066199 on 12 April 2023. Downloaded from http://bmjopen.bmj.com/ on April 27, 2023 by guest. Protected by copyright.

The manipulations will be carried out while the patient is in the supine position. The researcher/physiotherapist will passively lean over the patient to the side to be controlled, and the patient will be asked to place their hands behind their head (figure 3). The researcher will administer a posterior and inferior push on the opposite anterior superior iliac spine (ASIS) while passively rotating the subject on the side to be manipulated. The spinal manipulations will be repeated four times, with impulses oscillating between the right and left ASIS (twice on each side). In addition, we will administrate mobilisations (anterior–posterior central mobilisation), applied for 1 min in each lumbar vertebra (from L5 to L1), using grade I joint mobilisation (patients positioned in the ventral decubitus position).[61] The same protocol will be administered in all the sessions. The total SMT session will last approximately 10 min.

Five sessions of SMT will be administered to all participants (once per week). The physiotherapist will be blinded to group allocation. The researcher who will administer the manipulative therapy is a physiotherapist (doctorate in progress) with 5 years of clinical practice and certified in manual therapies (280 hours).

Sample size calculation

The study was designed to identify at least a two-point difference in pain intensity assessed by the NPRS across groups, with an expected SD of 2.5 points.[21] For GPE, a difference of two points on the scale, with 11 points ranging from −5 to +5.[40] The other specifications were an alpha of 5% and a power of 80%, with a total of 228 patients. However, assuming a 20% drop in follow-up, we will enrol 264 individuals (88 patients per group). G*Power was used to calculate sample size (G*Power V.3.0.10, University of Kiel, Germany).

Data analysis

The analysis will follow the intention-to-treat principles. An intention-to-treat analysis will be performed using the patient's last value gathered (last observation carried forward).

The mean effects of the interventions and the group differences for all outcomes (primary and secondary) will be calculated using linear mixed models, incorporating terms for the treatment groups, time (post-intervention and follow-up) and interaction terms (treatment subgroups and time). Secondary analysis will be carried out using multiple linear regression analysis and structural equation modelling to investigate the influence of baseline psychosocial variables (expectations, anxiety, depression, pain catastrophizing, pain self-efficacy, positive and negative affect) to predict the effect of the treatments when primary outcomes are considered. All data will be double-entered before the analysis. The analysis will follow the intention-to-treat principles. The statistician will receive coded data and will be blinded to the patients' allocation groups. The data collected will be stored and coded to protect patient confidentiality. To handle missing data, we will replace the value with the last observation carried forward. For all analyses, we will use the IBM SPSS software package, V.22 (IBM Corp, Armonk, New York, USA) and the significance level will be established at 0.05.

ETHICS AND DISSEMINATION

The current study was approved by the Ethics Committee of the Federal University of São Carlos (Process No. 52359521.1.0000.5504). Written informed consent will be obtained from all participants included in the study. The researchers will not pay the volunteers anything for their participation. Results will be presented at scientific meetings and published in international peer-reviewed journals. The study's funders (FAPESP) will receive summaries of the findings.

Author affiliations
[1]Department of Physical Therapy, UFSCar, Sao Carlos, Brazil
[2]Department of Orthopedic Surgery, Duke Clinical Research Institute, Durham, North Carolina, USA
[3]Department of Physiotherapy, Instituto Federal do Rio de Janeiro (IFRJ), Rio de Janeiro, Brazil, Rio de Janeiro, Brazil
[4]Departament of Physical Therapy, Federal University of Sao Carlos, Sao Carlos, Brazil
[5]University of Sao Paulo Faculty of Medicine of Ribeirao Preto, Ribeirao Preto, São Paulo, Brazil

Acknowledgements The authors would like to express sincere appreciation to the São Paulo Research Foundation – FAPESP (process number: 2019/22924-6 and 2019/23356-1) for financial support.

Contributors HCNC and DLCD are both assessors of the study, involved in data collection. HCNC is the assessor responsible for conducting the conditioning procedure and the researcher manager. DLCD is the blinded assessor, responsible to obtain the outcomes of the study. TCdL is the healthcare provider blinded to the verbal suggestion nature that each participant received. TCC is the chief researcher and responsible for the conceptualisation of the study and supervisor of the procedures. TCC, HCNC, FJJdR and SZG elaborated the design of the protocol. BESC is a researcher involved in the verbal suggestion phase of the study. All the authors reviewed the paper.

Funding This work was supported by the São Paulo Research Foundation – FAPESP (process number 2019/22924-6 and 2019/23356-1).

Competing interests None declared.

Patient and public involvement Patients and/or the public were not involved in the design, or conduct, or reporting, or dissemination plans of this research.

Patient consent for publication Consent obtained directly from patient(s).

Provenance and peer review Not commissioned; externally peer reviewed.

Supplemental material This content has been supplied by the author(s). It has not been vetted by BMJ Publishing Group Limited (BMJ) and may not have been peer-reviewed. Any opinions or recommendations discussed are solely those of the author(s) and are not endorsed by BMJ. BMJ disclaims all liability and responsibility arising from any reliance placed on the content. Where the content includes any translated material, BMJ does not warrant the accuracy and reliability of the translations (including but not limited to local regulations, clinical guidelines, terminology, drug names and drug dosages), and is not responsible for any error and/or omissions arising from translation and adaptation or otherwise.

Open access This is an open access article distributed in accordance with the Creative Commons Attribution Non Commercial (CC BY-NC 4.0) license, which permits others to distribute, remix, adapt, build upon this work non-commercially, and license their derivative works on different terms, provided the original work is properly cited, appropriate credit is given, any changes made indicated, and the use is non-commercial. See: http://creativecommons.org/licenses/by-nc/4.0/.

BMJ Open: first published as 10.1136/bmjopen-2022-066199 on 12 April 2023. Downloaded from http://bmjopen.bmj.com/ on April 27, 2023 by guest. Protected by copyright.

ORCID iD
Thaís Cristina Chaves http://orcid.org/0000-0002-6222-4961

REFERENCES

1 Wu A, March L, Zheng X, *et al*. Global low back pain prevalence and years lived with disability from 1990 to 2017: estimates from the global burden of disease study 2017. *Ann Transl Med* 2020;8:299.
2 GBD 2019 Diseases and Injuries Collaborators. Global burden of 369 diseases and injuries in 204 countries and territories, 1990-2019: a systematic analysis for the global burden of disease study 2019. *Lancet* 2020;396:1204–22.
3 Hartvigsen J, Hancock MJ, Kongsted A, *et al*. What low back pain is and why we need to pay attention. *Lancet* 2018;391:2356–67.
4 Maher C, Underwood M, Buchbinder R. Non-specific low back pain. *Lancet* 2017;389:736–47.
5 Deyo RA, Bryan M, Comstock BA, *et al*. Trajectories of symptoms and function in older adults with low back disorders. *Spine (Phila Pa 1976)* 2015;40:1352–62.
6 NICE guideline NG59. *2018 surveillance (exceptional review) of low back pain and sciatica in over 16s: assessment and management (NICE guideline NG59)*. London: National Institute for Health and Care Excellence (NICE), 2018.
7 Lin I, Wiles L, Waller R, *et al*. What does best practice care for musculoskeletal pain look like? Eleven consistent recommendations from high-quality clinical practice guidelines: systematic review. *Br J Sports Med* 2020;54:79–86.
8 George SZ, Fritz JM, Silfies SP, *et al*. Interventions for the management of acute and chronic low back pain: revision 2021. *J Orthop Sports Phys Ther* 2021;51:CPG1–60.
9 Rubinstein SM, de Zoete A, van Middelkoop M, *et al*. Benefits and harms of spinal manipulative therapy for the treatment of chronic low back pain: systematic review and meta-analysis of randomised controlled trials. *BMJ* 2019;364:l689.
10 Fillingim RB, Price DD. What is controlled for in placebo-controlled trials? *Mayo Clin Proc* 2005;80:1119–21.
11 Bialosky JE, Beneciuk JM, Bishop MD, *et al*. Unraveling the mechanisms of manual therapy: modeling an approach. *J Orthop Sports Phys Ther* 2018;48:8–18.
12 Bialosky JE, Bishop MD, Price DD, *et al*. The mechanisms of manual therapy in the treatment of musculoskeletal pain: a comprehensive model. *Man Ther* 2009;14:531–8.
13 Testa M, Rossettini G. Enhance placebo, avoid nocebo: how contextual factors affect physiotherapy outcomes. *Man Ther* 2016;24:65–74.
14 Rossettini G, Carlino E, Testa M. Clinical relevance of contextual factors as triggers of placebo and nocebo effects in musculoskeletal pain. *BMC Musculoskelet Disord* 2018;19:27.
15 Rossettini G, Camerone EM, Carlino E, *et al*. Context matters: the psychoneurobiological determinants of placebo, nocebo and context-related effects in physiotherapy. *Arch Physiother* 2020;10:11.
16 Peerdeman KJ, van Laarhoven AIM, Keij SM, *et al*. Relieving patients' pain with expectation interventions: a meta-analysis. *Pain* 2016;157:1179–91.
17 Finniss D, Nicholas M, Brooker C, *et al*. Magnitude, response, and psychological determinants of placebo effects in chronic low-back pain: a randomised, double-blinded, controlled trial. *Pain Rep* 2019;4:e744.
18 Benedetti F. Mechanisms of placebo and placebo-related effects across diseases and treatments. *Annu Rev Pharmacol Toxicol* 2008;48:33–60.
19 Atlas LY, Wager TD. How expectations shape pain. *Neurosci Lett* 2012;520:140–8.
20 Verbeek J, Sengers MJ, Riemens L, *et al*. Patient expectations of treatment for back pain: a systematic review of qualitative and quantitative studies. *Spine (Phila Pa 1976)* 2004;29:2309–18.
21 Malfliet A, Lluch Girbés E, Pecos-Martin D, *et al*. The influence of treatment expectations on clinical outcomes and cortisol levels in patients with chronic neck pain: an experimental study. *Pain Pract* 2019;19:370–81.
22 Bingel U, Wanigasekera V, Wiech K, *et al*. The effect of treatment expectation on drug efficacy: imaging the analgesic benefit of the opioid remifentanil. *Sci Transl Med* 2011;3:70.
23 Colloca L, Tinazzi M, Recchia S, *et al*. Learning potentiates neurophysiological and behavioral placebo analgesic responses. *Pain* 2008;139:306–14.
24 Kong J, Spaeth R, Cook A, *et al*. Are all placebo effects equal? Placebo pills, sham acupuncture, cue conditioning and their association. *PLoS One* 2013;8:e67485.
25 Kong J, Wang Z, Leiser J, *et al*. Enhancing treatment of osteoarthritis knee pain by boosting expectancy: a functional neuroimaging study. *Neuroimage Clin* 2018;18:325–34.
26 Bąbel P. Classical conditioning as a distinct mechanism of placebo effects. *Front Psychiatry* 2019;10:449.
27 Bialosky JE, Bishop MD, Robinson ME, *et al*. The influence of expectation on spinal manipulation induced hypoalgesia: an experimental study in normal subjects. *BMC Musculoskelet Disord* 2008;9:19.
28 Sherriff B, Clark C, Killingback C, *et al*. Impact of contextual factors on patient outcomes following conservative low back pain treatment: systematic review. *Chiropr Man Therap* 2022;30:20.
29 Juszczak E, Altman DG, Hopewell S, *et al*. Reporting of multi-arm parallel-group randomized trials: extension of the CONSORT 2010 statement. *JAMA* 2019;321:1610–20.
30 Chan A-W, Tetzlaff JM, Altman DG, *et al*. SPIRIT 2013 statement: defining standard protocol items for clinical trials. *Ann Intern Med* 2013;158:200–7.
31 Zajacova A, Grol-Prokopczyk H, Zimmer Z. Pain trends among American adults, 2002-2018: patterns, disparities, and correlates. *Demography* 2021;58:711–38.
32 Chiarotto A, Maxwell LJ, Ostelo RW, *et al*. Measurement properties of visual analogue scale, numeric rating scale, and pain severity subscale of the brief pain inventory in patients with low back pain: a systematic review. *J Pain* 2019;20:245–63.
33 Vibe Fersum K, O'Sullivan P, Skouen JS, *et al*. Efficacy of classification-based cognitive functional therapy in patients with non-specific chronic low back pain: a randomized controlled trial. *Eur J Pain* 2013;17:916–28.
34 Whitman JM. Pregnancy, low back pain, and manual physical therapy interventions. *J Orthop Sports Phys Ther* 2002;32:314–7.
35 Clark JD, Bair MJ, Belitskaya-Lévy I, *et al*. Sequential and comparative evaluation of pain treatment effectiveness response (SCEPTER), a pragmatic trial for conservative chronic low back pain treatment. *Contemp Clin Trials* 2023;125:107041.
36 Puentedura EL. Spinal manipulation. In: Giangarra CE, Manske RC, eds. *Clinical orthopaedic rehabilitation: a team approach*. Fourth edition. Philadelphia, PA: Elsevier, 2018.
37 Medoc®. Israel website. Available: https://www.manualslib.com/manual/1580151/Medoc-Q-Sense.html?page=2) [Accessed 29 Dec 2022].
38 Costa LOP, Maher CG, Latimer J, *et al*. Clinimetric testing of three self-report outcome measures for low back pain patients in Brazil: which one is the best? *Spine (Phila Pa 1976)* 2008;33:2459–63.
39 Salaffi F, Stancati A, Silvestri CA, *et al*. Minimal clinically important changes in chronic musculoskeletal pain intensity measured on a numerical rating scale. *Eur J Pain* 2004;8:283–91.
40 Kamper SJ, Maher CG, Mackay G. Global rating of change scales: a review of strengths and weaknesses and considerations for design. *J Man Manip Ther* 2009;17:163–70.
41 Vigatto R, Alexandre NMC, Correa Filho HR. Development of a Brazilian Portuguese version of the oswestry disability index: cross-cultural adaptation, reliability, and validity. *Spine (Phila Pa 1976)* 2007;32:481–6.
42 Ostelo RWJG, Deyo RA, Stratford P, *et al*. Interpreting change scores for pain and functional status in low back pain: towards international consensus regarding minimal important change. *Spine (Phila Pa 1976)* 2008;33:90–4.
43 Younger J, Gandhi V, Hubbard E, *et al*. Development of the Stanford expectations of treatment scale (sets): a tool for measuring patient outcome expectancy in clinical trials. *Clin Trials* 2012;9:767–76.
44 Eklund A, De Carvalho D, Pagé I, *et al*. Expectations influence treatment outcomes in patients with low back pain. A secondary analysis of data from a randomized clinical trial. *Eur J Pain* 2019;23:1378–89.
45 de Lima Osório F, Vilela Mendes A, Crippa JA, *et al*. Study of the discriminative validity of the PHQ-9 and PHQ-2 in a sample of Brazilian women in the context of primary health care. *Perspect Psychiatr Care* 2009;45:216–27.
46 Moreno-Agostino D, Chua KC, Peters TJ, *et al*. Psychometric properties of the PHQ-9 measure of depression among Brazilian older adults. *Aging Ment Health* 2022;26:2285–90.
47 Moreno AL, DeSousa DA, Souza AMFLP, *et al*. Factor structure, reliability, and item parameters of the Brazilian-portuguese version of the GAD-7 questionnaire. *Temas Psicol* 2016;24:367–76.
48 Siqueira FB, Teixeira-Salmela LF, Magalhães L de C. Análise das propriedades psicométricas dA versão brasileira dA escala Tampa de cinesiofobia. *Acta Ortop Bras* 2007;15:19–24.
49 Sehn F, Chachamovich E, Vidor LP, *et al*. Cross-Cultural adaptation and validation of the Brazilian Portuguese version of the pain catastrophizing scale. *Pain Med* 2012;13:1425–35.

BMJ Open: first published as 10.1136/bmjopen-2022-066199 on 12 April 2023. Downloaded from http://bmjopen.bmj.com/ on April 27, 2023 by guest. Protected by copyright.

50 Watson D, Clark LA, Tellegen A. Development and validation of brief measures of positive and negative affect: the PANAS scales. *J Pers Soc Psychol* 1988;54:1063–70.
51 Pires P, Filgueiras A, Ribas R, *et al*. Positive and negative affect schedule: psychometric properties for the Brazilian Portuguese version. *Span J Psychol* 2013;16:E58.
52 Sardá J, Nicholas MK, Pimenta CAM, *et al*. Pain-related self-efficacy beliefs in a brazilian chronic pain patient sample: a psychometric analysis. *Stress and Health* 2007;23:185–90.
53 Gallagher L, McAuley J, Moseley GL. A randomized-controlled trial of using a book of metaphors to reconceptualize pain and decrease catastrophizing in people with chronic pain. *Clin J Pain* 2013;29:20–5.
54 Chaves TC, Stanton TR, Grant A, *et al*. Imprecise visual feedback about hand location increases a classically conditioned pain expectancy effect. *J Pain* 2021;22:748–61.
55 Chaves TC, Dach F, Florencio LL, *et al*. Concomitant migraine and temporomandibular disorders are associated with higher heat pain hyperalgesia and cephalic cutaneous allodynia. *Clin J Pain* 2016;32:882–8.
56 Gracely RH, McGrath P, Dubner R. Ratio scales of sensory and affective verbal pain descriptors. *Pain* 1978;5:5–18.
57 Fritz JM, Delitto A, Erhard RE. Comparison of classification-based physical therapy with therapy based on clinical practice guidelines for patients with acute low back pain: a randomized clinical trial. *Spine (Phila Pa 1976)* 2003;28:1363–71;
58 Egorova N, Park J, Orr SP, *et al*. Not seeing or feeling is still believing: conscious and non-conscious pain modulation after direct and observational learning. *Sci Rep* 2015;5:16809.
59 Flynn T, Fritz J, Whitman J, *et al*. A clinical prediction rule for classifying patients with low back pain who demonstrate short-term improvement with spinal manipulation. *Spine (Phila Pa 1976)* 2002;27:2835–43.
60 George SZ, Bishop MD, Bialosky JE, *et al*. Immediate effects of spinal manipulation on thermal pain sensitivity: an experimental study. *BMC Musculoskelet Disord* 2006;7:68.
61 Maitland G, Hengeveld E. *Maitland's vertebral manipulation*. 7th ed. London: Elsevier, 2005.

BMJ Open: first published as 10.1136/bmjopen-2022-066199 on 12 April 2023. Downloaded from http://bmjopen.bmj.com/ on April 27, 2023 by guest. Protected by copyright.
