# Supplementary material for: Effects of Hidden Conditioning and Positive Expectations on Pain Intensity Following Spinal Manipulative Therapy: A Three‐Arm Randomized Controlled Trial
Source: Eur J Pain. 2026 Oct 12;30(9):e70414. doi: 10.1002/ejp.70414 (PMC13656883; doi:10.1002/ejp.70414)
Supplement: Supplementary file 3 [file EJP-30-0-s002.docx]

*Pain intensity post-treatment/Disability/ GPE of improvement*

*Positive/Negative Expectation baseline*

b

a

*Pain intensity*

c’

*Kinesiophobia/pain catastrophizing/pain self-efficacy/ depression/anxiety assessed at baseline*

*Pain intensity post-treatment/Disability/ GPE of improvement*

*Pain intensity*

b

a

c’

**Figure S1.** The path diagrams illustrate the directed acyclic graphs (DAGs) for the mediation analysis adopted in the study. The mediation variable is highlighted in the gray rectangle. The outcomes (black rectangle) were assessed at 1-month and 3-month follow-ups. The DE is the effect of the independent variable on the outcome (dependent variable), excluding the influence of the mediator (letter c’). The MOE is the direct effect of the mediator on the outcome (letter b), the IVME is the effect observed between the independent variable and the mediator (letter a), and IE is the effect of the independent variable on the outcome (dependent variable) mediated by the positive/negative expectation or psychosocial variables (obtained by multiplying a*b).

Legend:

a (Independent Variable-Mediator Effect - IVME) = direct relationship between the independent variable and the mediator

b = (Mediator-Outcome Effect – MOE) direct relationship between the outcome (dependent variable) and the mediator

c’ (Direct Effect – IE) = direct effect between the independent variable and the dependent variable, excluding the effect of the mediator

c = a*b (Indirect Effect - IE) = the indirect effect between the dependent and independent variable mediated by a third variable (mediator variable)

Total effect (c = c’ + a*b; TE = DE + IE)

GPE = Global Perceived Effect
