# Supplementary material for: Effects of Hidden Conditioning and Positive Expectations on Pain Intensity Following Spinal Manipulative Therapy: A Three‐Arm Randomized Controlled Trial
Source: Eur J Pain. 2026 Oct 12;30(9):e70414. doi: 10.1002/ejp.70414 (PMC13656883; doi:10.1002/ejp.70414)
Supplement: Supplementary file 4 [file EJP-30-0-s003.docx]

**Positive verbal suggestion – C&PEG (conditioning and positive expectation group) and PEG (positive expectation group)**

“Hello, how are you doing? My name is Thais Chaves. I am a physiotherapist, University professor, and researcher at the physiotherapy department of the Federal University of São Carlos – UFSCAR.

My research focuses on understanding the best treatment options for patients who have chronic pain, persistent pain. Today, we are going to talk about the treatment that will be administered in this study, which is manual therapy. Do you know what manual therapy is?

Manual therapy is a treatment in which the physiotherapist uses the hands to apply different techniques, like the one you can see in the video, by my side. In this study, the manual therapy technique will be applied to the lower back, which is the lumbar spine. One of the effects of manual therapy is to improve joint movements, and it helps to promote pain relief.

Manual therapy is a treatment commonly used by physiotherapists. Scientific studies have shown that manual therapy is effective in reducing the pain in patients with low back pain, and it also helps patients to resume the activities impaired due to pain.

Then, it is likely you will perceive an improvement in your back pain after manual therapy. Thank you for your attention and for participating in this study.”

**Neutral verbal suggestion – NEUG (neutral expectation group)**

“Hello, how are you doing? My name is Thais Chaves. I am a physiotherapist, University professor, and researcher at the physiotherapy department of the Federal University of São Carlos – UFSCAR.

My research focuses on understanding the best treatment options for patients who have chronic pain, persistent pain. Today, we are going to talk about the treatment that will be administered in this study, which is recognized as manual therapy. Do you know what manual therapy is?

Manual therapy is a treatment in which the physiotherapist uses their hands to apply different techniques, like the one you can see in the video, by my side. In this study, the manual therapy technique will be applied to the lower back, which is called the lumbar spine. One of the effects of manual therapy is to improve joint movements, which can contribute to pain relief.

Manual therapy is a treatment commonly used by physiotherapists. For some patients, Manual Therapy can promote pain relief and also help them to resume activities impaired due to pain. But for some patients, manual therapy may not be effective.

Thus, it is possible that after treatment with manual therapy, you will perceive an improvement in your back pain or no improvement.

Thank you for your kind attention and for participating in this study.”
